# Supplementary material for: Exogenous application of the apocarotenoid retinaldehyde negatively regulates auxin-mediated root growth
Source: Plant Physiol. 2024 Aug 9;196(2):1659–73. doi: 10.1093/plphys/kiae405 (PMC11483604; doi:10.1093/plphys/kiae405)
Supplement: kiae405_Supplementary_Data [file kiae405_supplementary_data.zip › plant physio XU KANG et al 2024 Supplemental Video S1 Legend revision2.docx]

**Supplementary Movie Legend**

Supplementary Video S1. The *DR5* bioluminescence video under fluridone treatment in WT and *mPDS*. Five-day-old plants were transferred to new medium with or without 800 nM fluridone. Scale bar = 5 mm.
