# Supplementary material for: Exogenous application of the apocarotenoid retinaldehyde negatively regulates auxin-mediated root growth
Source: Plant Physiol. 2024 Aug 9;196(2):1659–73. doi: 10.1093/plphys/kiae405 (PMC11483604; doi:10.1093/plphys/kiae405)
Supplement: kiae405_Supplementary_Data [file kiae405_supplementary_data.zip › PP2024RA00788DR1_Supplemental_Figures_1_17.pdf]

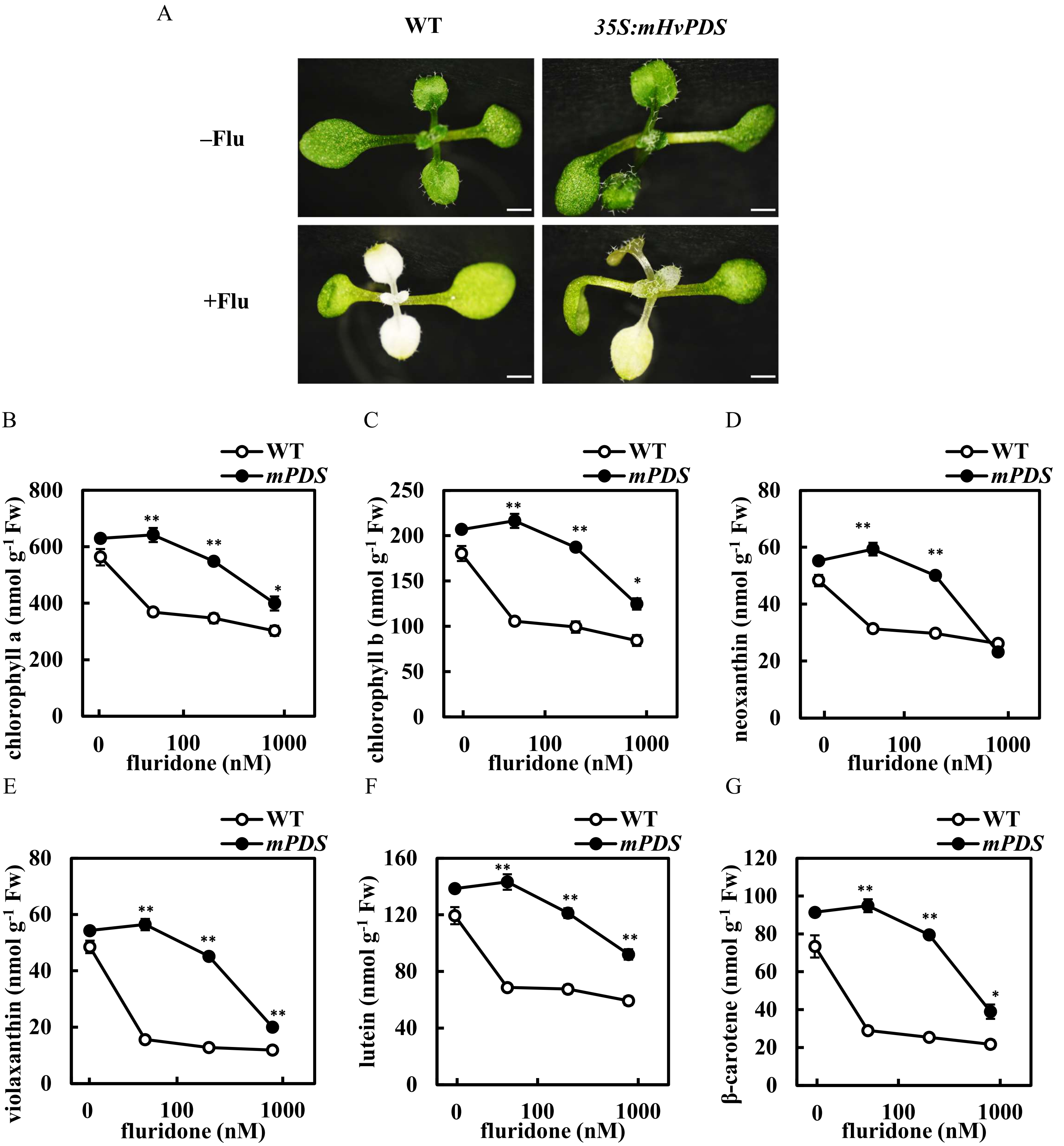


Supplementary Figure S1. Fluridone reduces chlorophyll and carotenoid contents, and *mPDS* plants are resistant to it. A–G) Five-day-old plants of wild-type (WT) and *mPDS* were transferred to medium with or without 800 nM fluridone. (A) Leaf pigment bleaching effect of WT and *mPDS* by fluridone. Photographs were captured at 3 d after transfer (dat). Scale bar = 5 mm. Chlorophyll a (B), chlorophyll b (C), neoxanthin (D), violaxanthin (E), lutein (F) and β-carotene (G) contents in the whole plants of WT (hollow circle) and *mPDS* (solid circle) were quantified at 2 dat. Data represent the means ± SE from 3 biological replicates. *Significant differences compared to WT (*: $P < 0.05$, **: $P < 0.01$; Student's *t*-test).

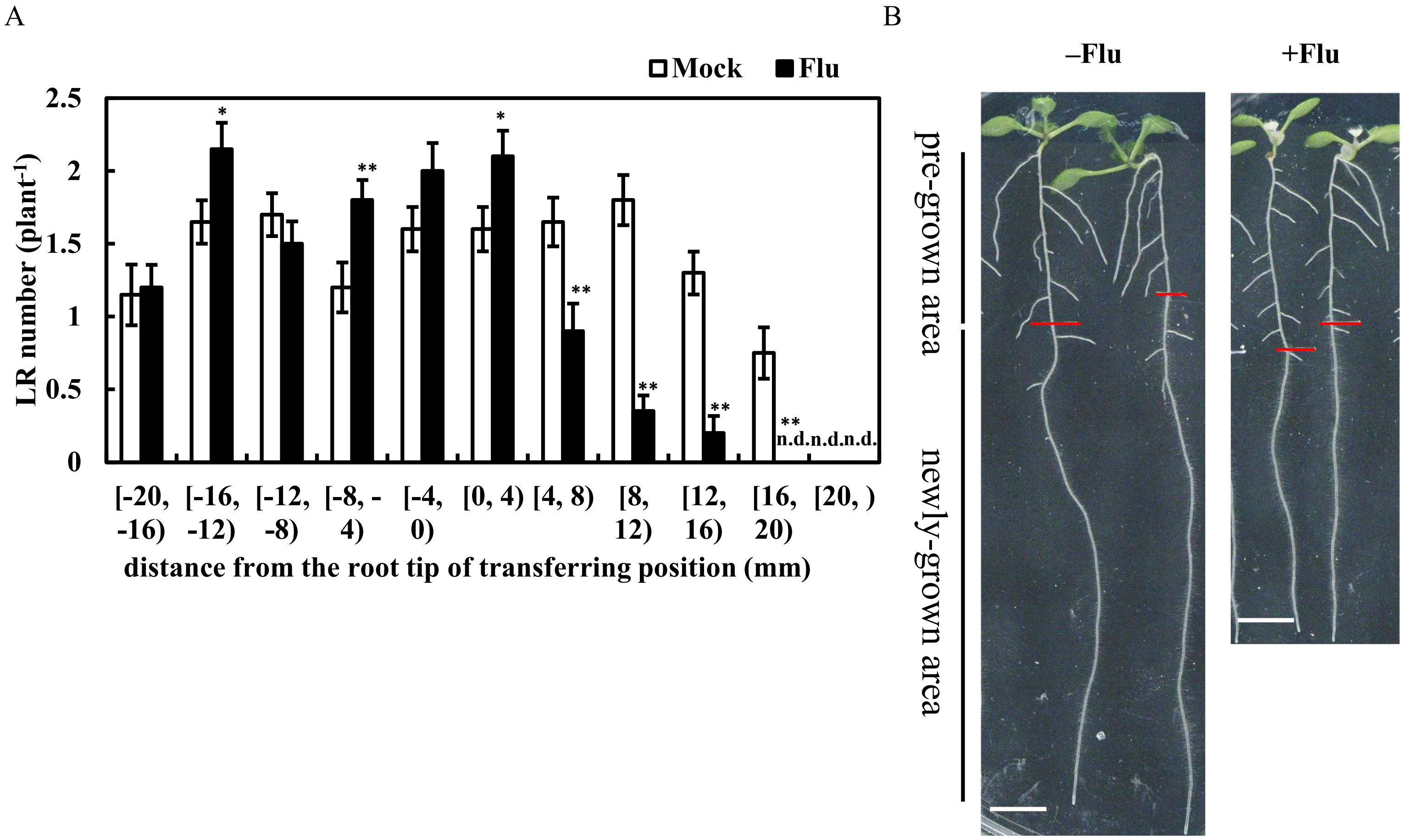


Supplementary Figure S2. Fluridone induces LR formation in the pre-grown areas of PR but severely reduces it in the newly grown areas of PR. A, B) Five-day-old plants were transferred to medium with or without 800 nM fluridone. The number of lateral roots (LRs) was counted at 4 d after transfer (dat). The root tips of the transferring positions were positioned as 0. The pre-grown root was positioned as < 0. The newly grown root was positioned as > 0. Photographs were captured at 4 dat. Scale bar = 5 mm. Red lines indicate the root tips of the transferring position. Data represent the means ± SE from 20 seedlings. *Significant differences compared to mock-treated plants (*: $P < 0.05$, **: $P < 0.01$; Student's *t*-test). n.d. indicates no detectable data.

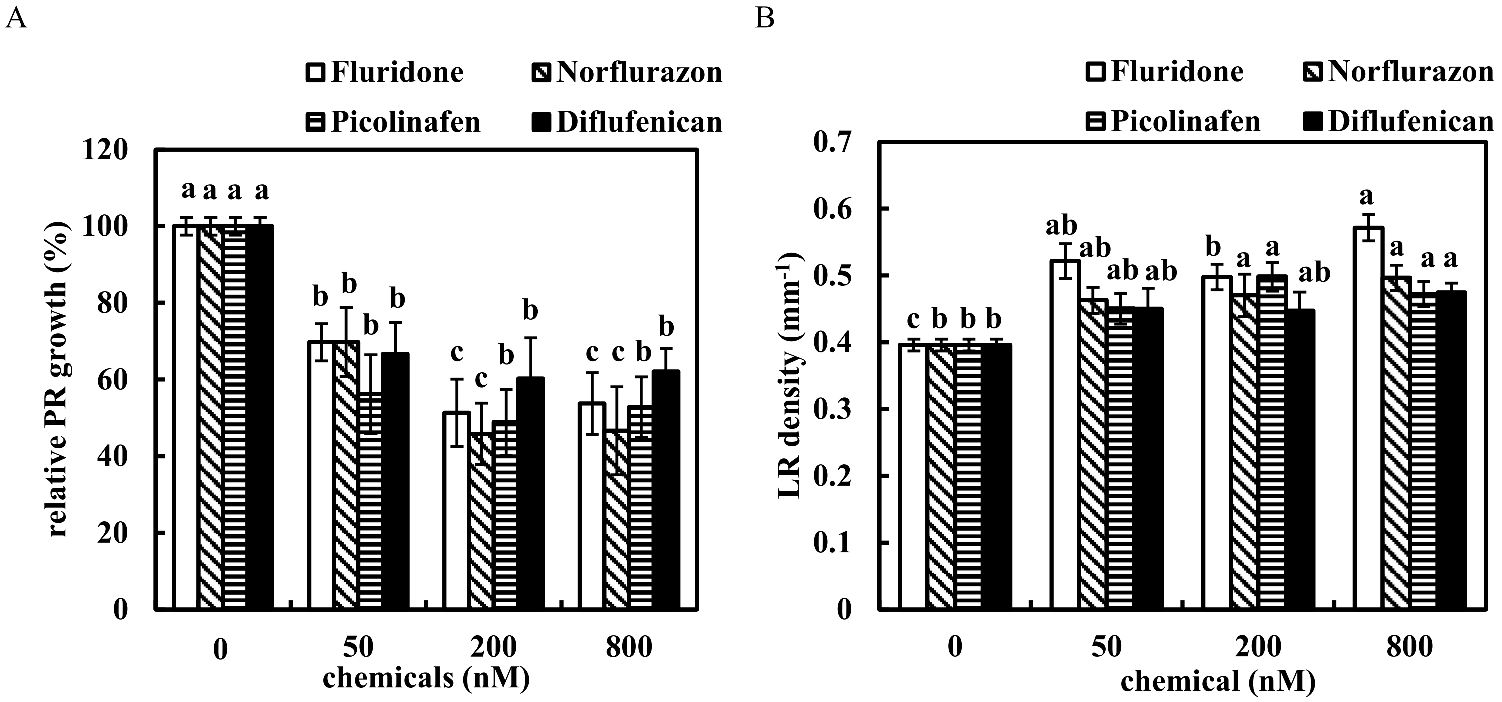


Supplementary Figure S3. PDS inhibitors exhibit a fluridone-like effect on PR suppression and LR induction. A, B) Five-day-old plants were transferred to medium with or without inhibitors. The relative primary root (PR) growth rate compared to mock-treated plants (A) was analyzed from 2 to 3 d after transfer (dat). The lateral root (LR) density (B) was counted at 3 dat. The bars indicate fluridone (white), norflurazon (diagonal striped black), picolinafen (horizontal striped black), and diflufenican (black). Data represent the means ± SE from 8 to 14 seedlings. Different lowercase letters above the bars indicate significant differences at $P < 0.05$ (one-way ANOVA following Tukey-Kramer test).

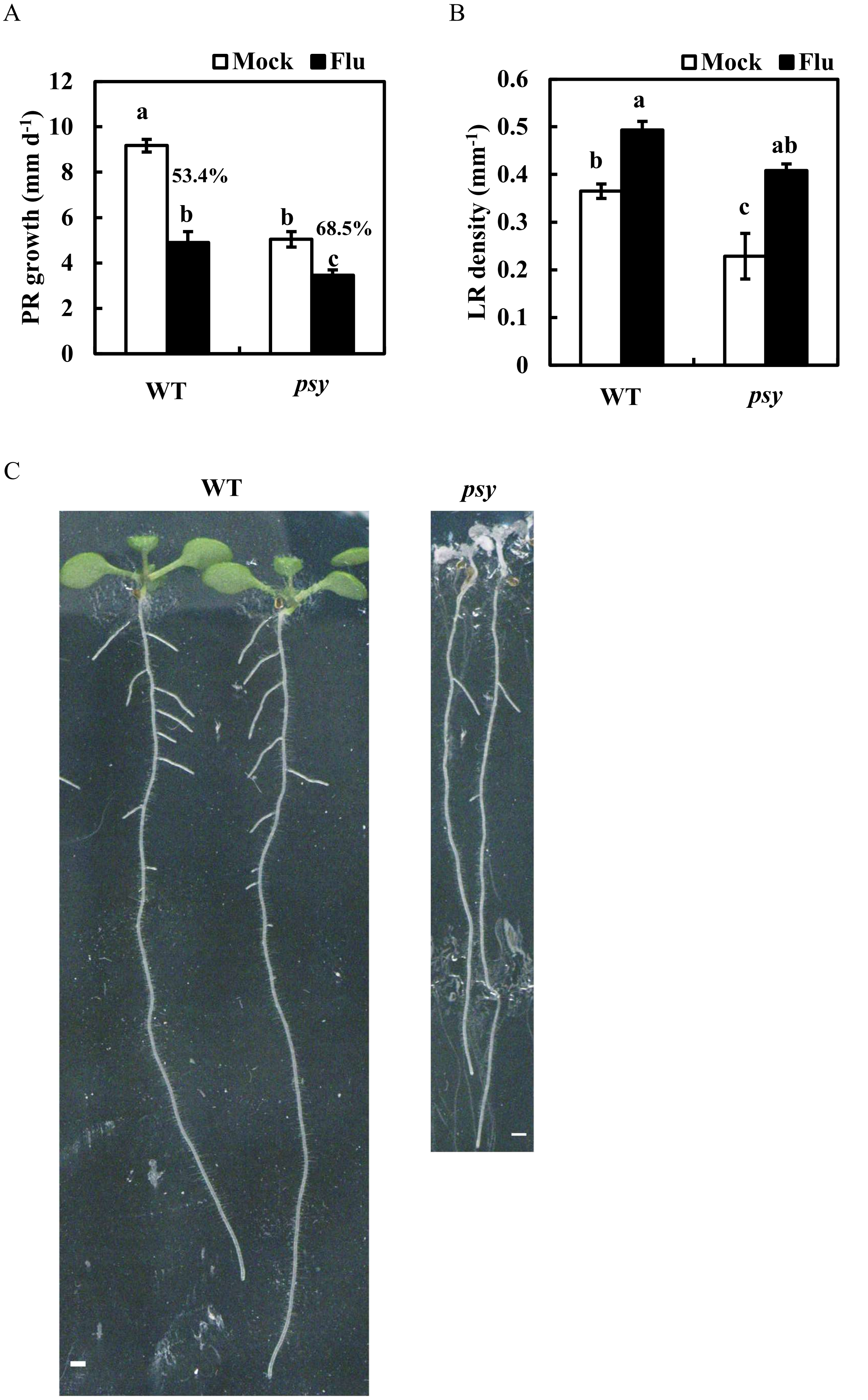


Supplementary Figure S4. The phenotypes and fluridone resistance of the *psy* mutant. A, B) Five-day-old plants of wild-type (WT) and *psy* were transferred to medium with or without 800 nM fluridone. The primary root (PR) growth rate (A) was analyzed from 2 to 3 d after transfer (dat). The lateral root (LR) density (B) was counted at 3 dat. C) The pictures of WT and *psy* mutant were captured at 8 d after germination (without transfer). Scale bar = 1 mm. The percentage in (A) means the relative PR growth rate compared to mock-treated plants. Data represent the means ± SE from 10 seedlings in (A, B). Different lowercase letters above the bars indicate significant differences at $P < 0.05$ (one-way ANOVA following Tukey-Kramer test).

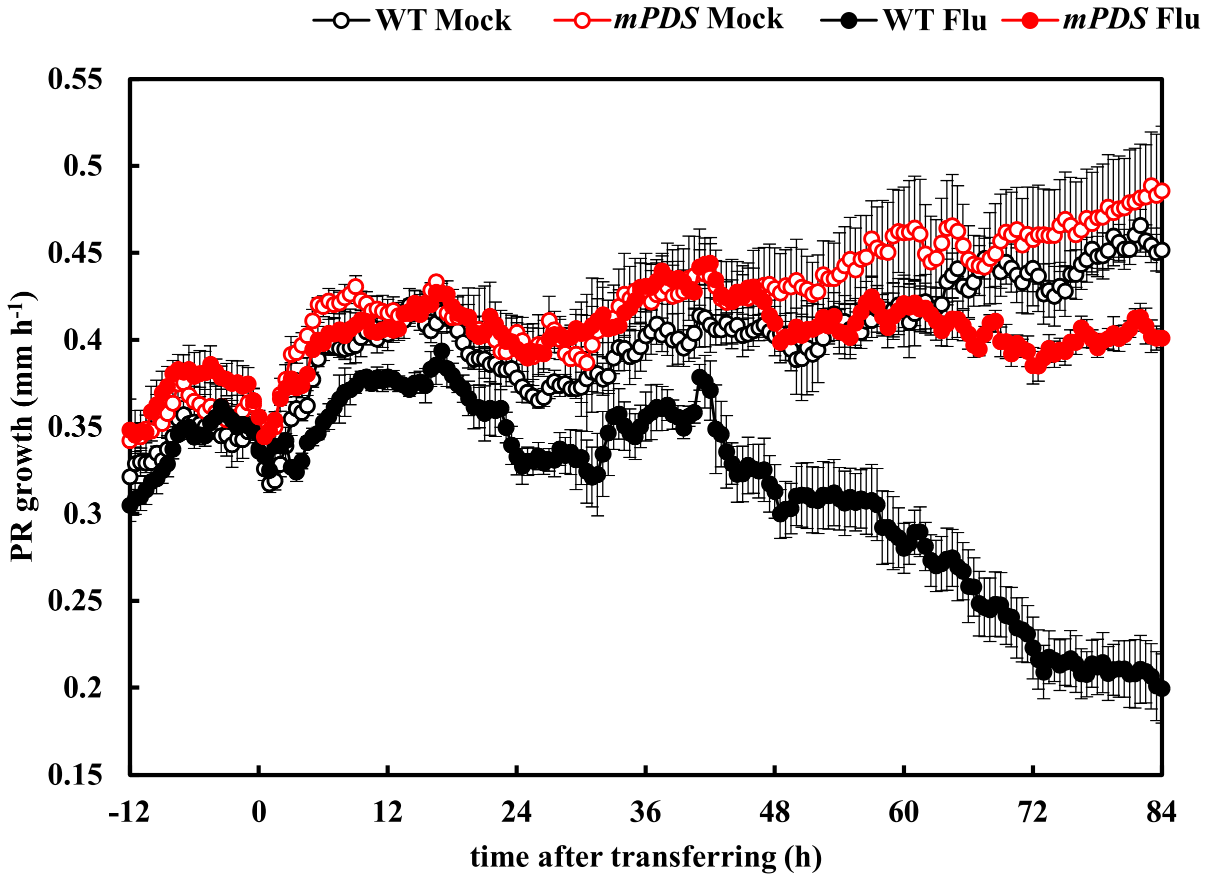


Supplementary Figure S5. The kinetic growth rate of PRs in WT and *mPDS* after fluridone treatment. Four-day-old plants of wild-type (WT) and *mPDS* were transferred to mock medium for a 1 d preincubation. Then plants were transferred to new medium with or without 800 nM fluridone at time 0. The primary root (PR) growth rate was measured from preincubation at an interval of 30 min, which was indicated as WT mock-treated plants (hollow black), WT fluridone-treated plants (solid black), *mPDS* mock-treated plants (hollow red), and *mPDS* fluridone-treated plants (solid red). Data represent the means ± SE from 5 seedlings.

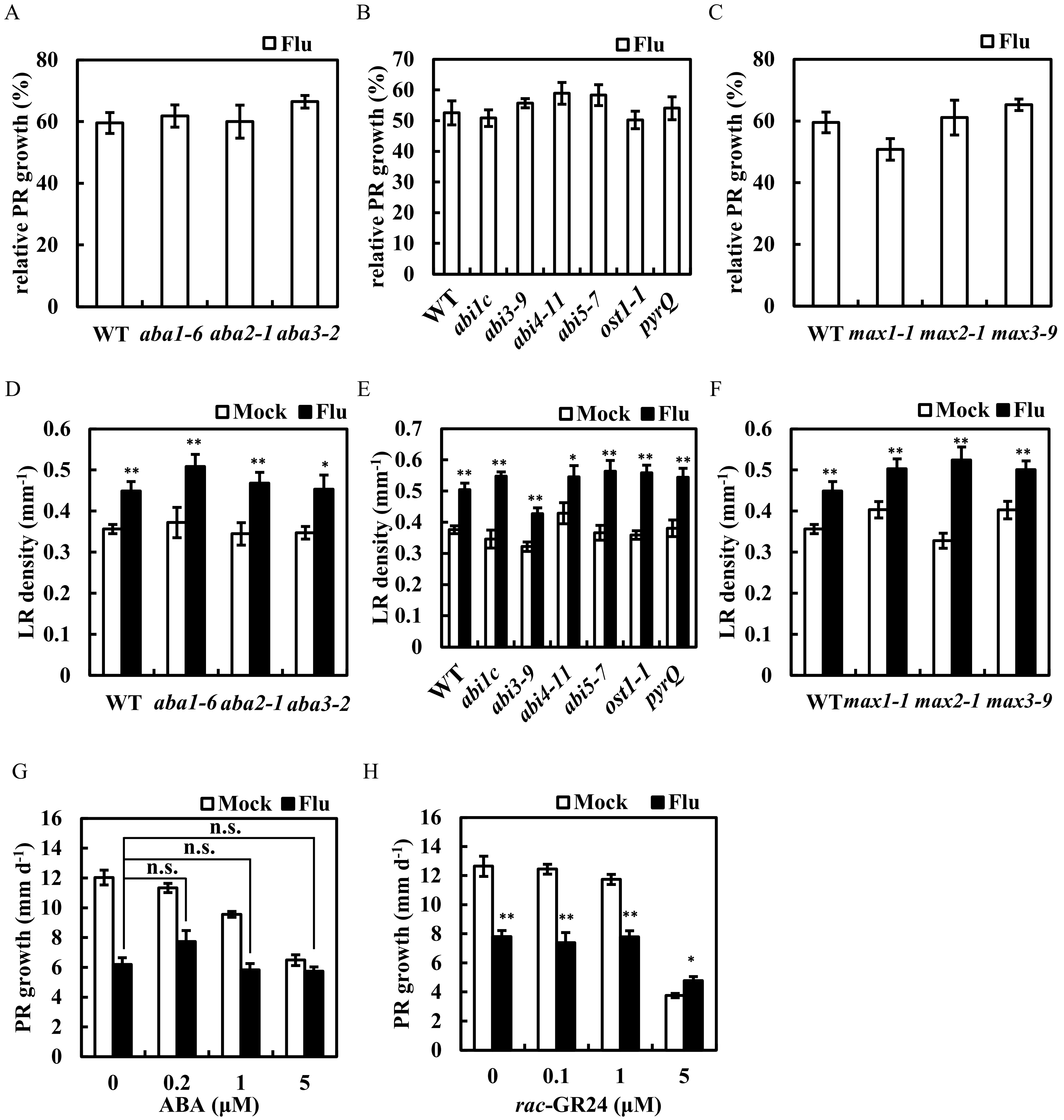


Supplementary Figure S6. ABA and SLs are not involved in fluridone-mediated regulation of root architecture. A–F) The resistance to fluridone in ABA-related and SL-related mutants. Five-day-old plants were transferred to medium with or without 800 nM fluridone. (A, D) ABA biosynthesis mutants. (B, E) ABA signaling mutants. (C, F) SL biosynthesis and signaling mutants. The relative primary root (PR) growth rate compared to mock-treated plants was analyzed from 2 to 3 d after transfer (dat). The lateral root (LR) density was counted at 3 dat. G, H) Five-day-old plants were transferred to medium with or without fluridone and ABA (G) or *rac*-GR24 (H). The PR growth rate was analyzed from 2 to 3 dat. Data represent the means ± SE from 10 seedlings. *Significant differences compared to mock-treated plants in (D, E, F, H) (*: $P < 0.05$, **: $P < 0.01$; Student's *t*-test). No significant difference was detected between WT and mutants in (A–C) (one-way ANOVA following Tukey-Kramer test). n.s. indicates no significant difference was detected between non ABA-treated and ABA-treated plants in (G) (Student's *t*-test).

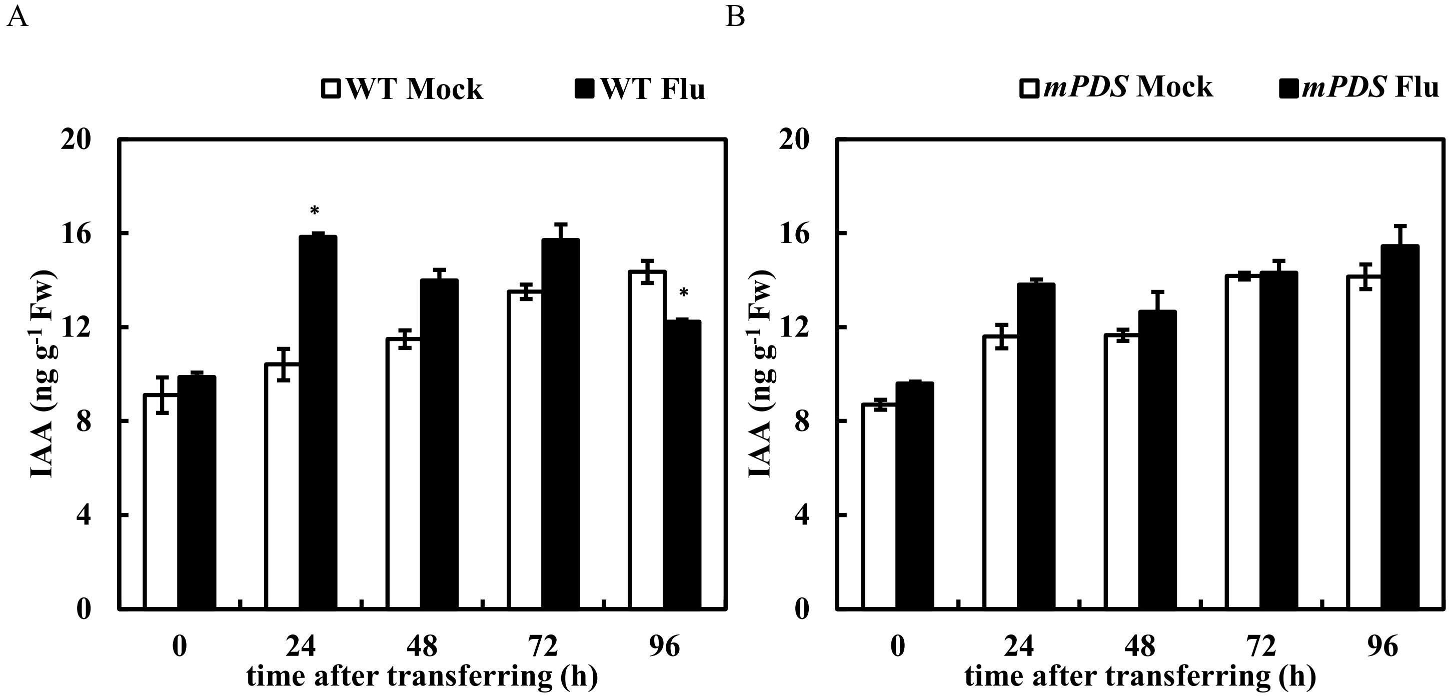


Supplementary Figure S7. Long-period change of auxin levels by fluridone. A, B) Five-day-old plants were transferred to medium with or without 800 nM fluridone at time 0. The IAA contents in the roots of wild-type (WT) (A) and *mPDS* (B) were measured at the indicated time points. Data represent the means ± SE from 3 biological replicates. *Significant differences compared to mock plants (*: $P < 0.05$; Student's *t*-test). No significant difference was detected between mock-treated and fluridone-treated plants in (B) (Student's *t*-test).

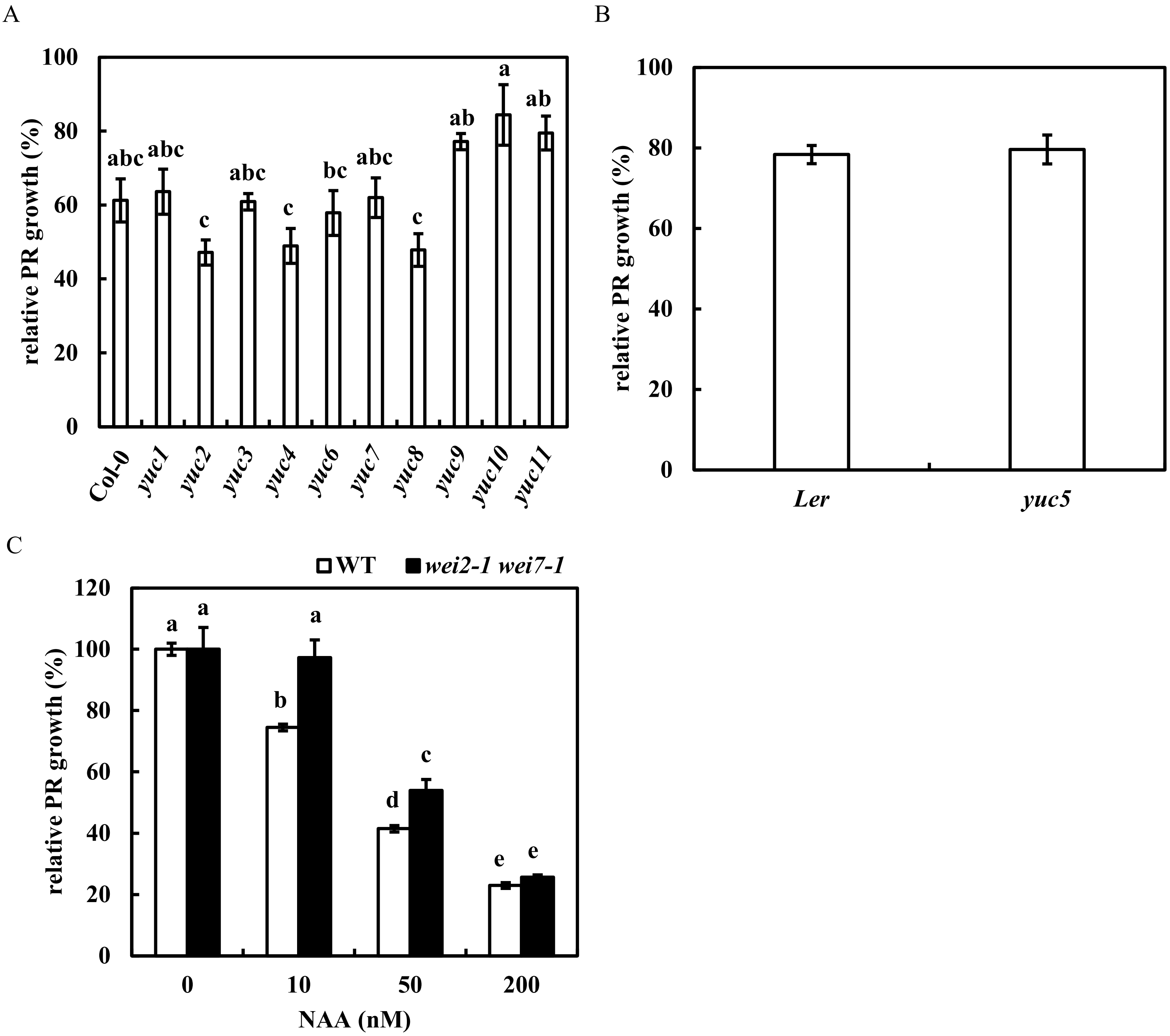


Supplementary Figure S8. The *yuc* single mutants have weak or no resistance to fluridone, and *wei2-1 wei7-1* shows resistance to low dose of exogenous NAA. A, B) Five-day-old plants were transferred to medium with or without 800 nM fluridone. (A) The *yuc* mutants in a Columbia-0 (Col-0) background. (B) The *yuc* mutant in a Landsberg *erecta* (*Ler*) background. C) Five-day-old plants of wild-type (WT) and *wei2-1 wei7-1* were transferred to medium with or without NAA. The relative primary root (PR) growth rate compared to mock-treated plants was analyzed from 2 to 3 d after transfer (dat). The relative PR growth rate in (A–C) was compared to mock-treated plants whose root growth rate is listed in Supplementary Table S3. Data represent the means ± SE from 8-10 seedlings. Different lowercase letters above the bars in (A, C) indicate significant differences at $P < 0.05$ (one-way ANOVA following Tukey-Kramer test). No significant difference was detected between *Ler* and *yuc5* in (B) (Student's *t*-test).

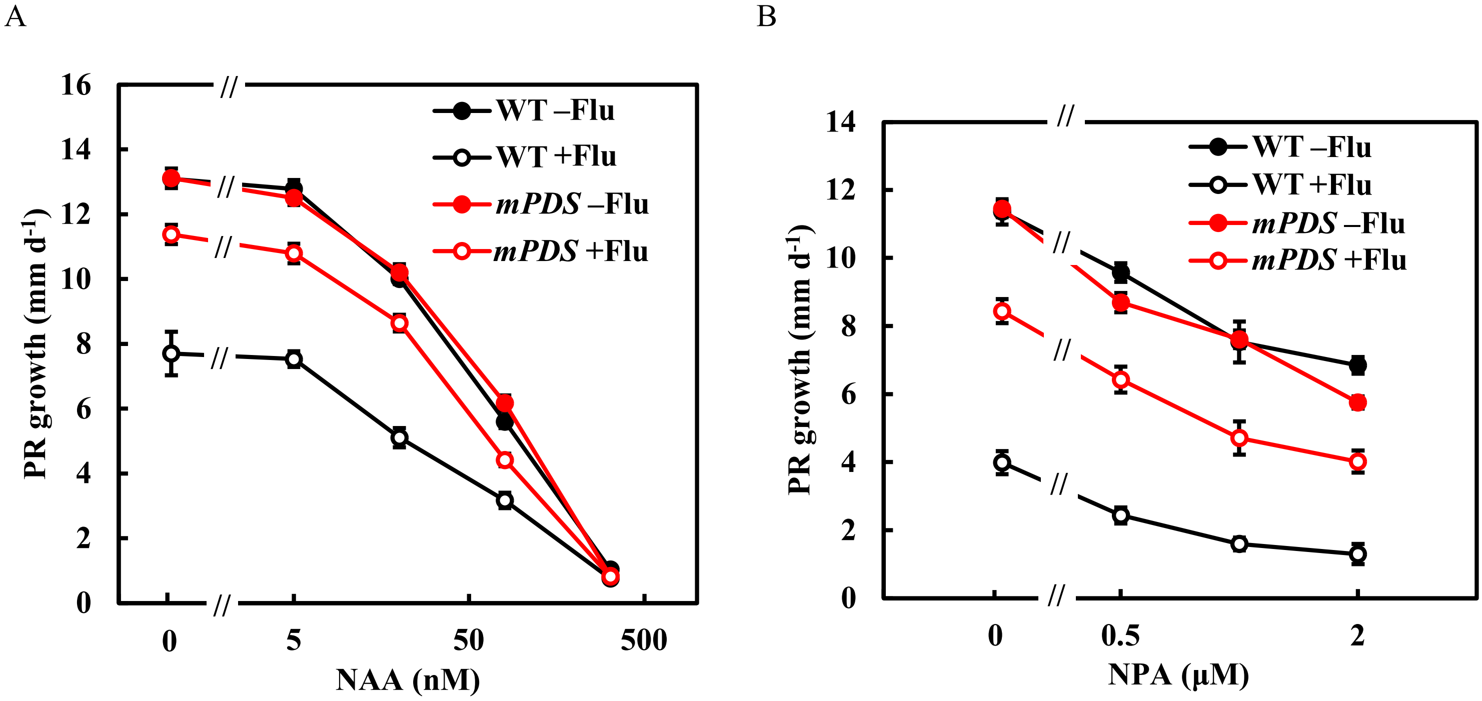


Supplementary Figure S9. Exogenous auxin and NPA suppress PR growth in an additive manner with fluridone. A, B) Five-day-old plants of wild-type (WT) and *mPDS* were transferred to medium with or without 800 nM fluridone and NAA (A) or NPA (B). The primary root (PR) growth rate was analyzed from 2 to 3 d after transfer (dat) in (A) or 3 to 4 dat in (B). Data represent the means ± SE from 8 seedlings. For (A), the relative PR growth of fluridone-treated plants to non-fluridone-treated plants is as follows: 58.8% (WT, 0 nM NAA), 86.8% (*mPDS*, 0 nM NAA), 58.9% (WT, 5 nM NAA), 86.3% (*mPDS*, 5 nM NAA), 51.0% (WT, 20 nM NAA), 84.7% (*mPDS*, 20 nM NAA), 47.2% (WT, 80 nM NAA), 79.9% (*mPDS*, 80 nM NAA), 72.9% (WT, 320 nM NAA) and 97.8% (*mPDS*, 320 nM NAA). For (B), the relative PR growth of fluridone-treated plants to non-fluridone-treated plants is as follows: 35.1% (WT, 0 µM NPA), 73.8% (*mPDS*, 0 µM NPA), 25.5% (WT, 0.5 µM NPA), 73.9% (*mPDS*, 0.5 µM NPA), 21.2% (WT, 1 µM NPA), 61.9% (*mPDS*, 1 µM NPA), 19.0% (WT, 2 µM NPA) and 69.8% (*mPDS*, 2 µM NPA).

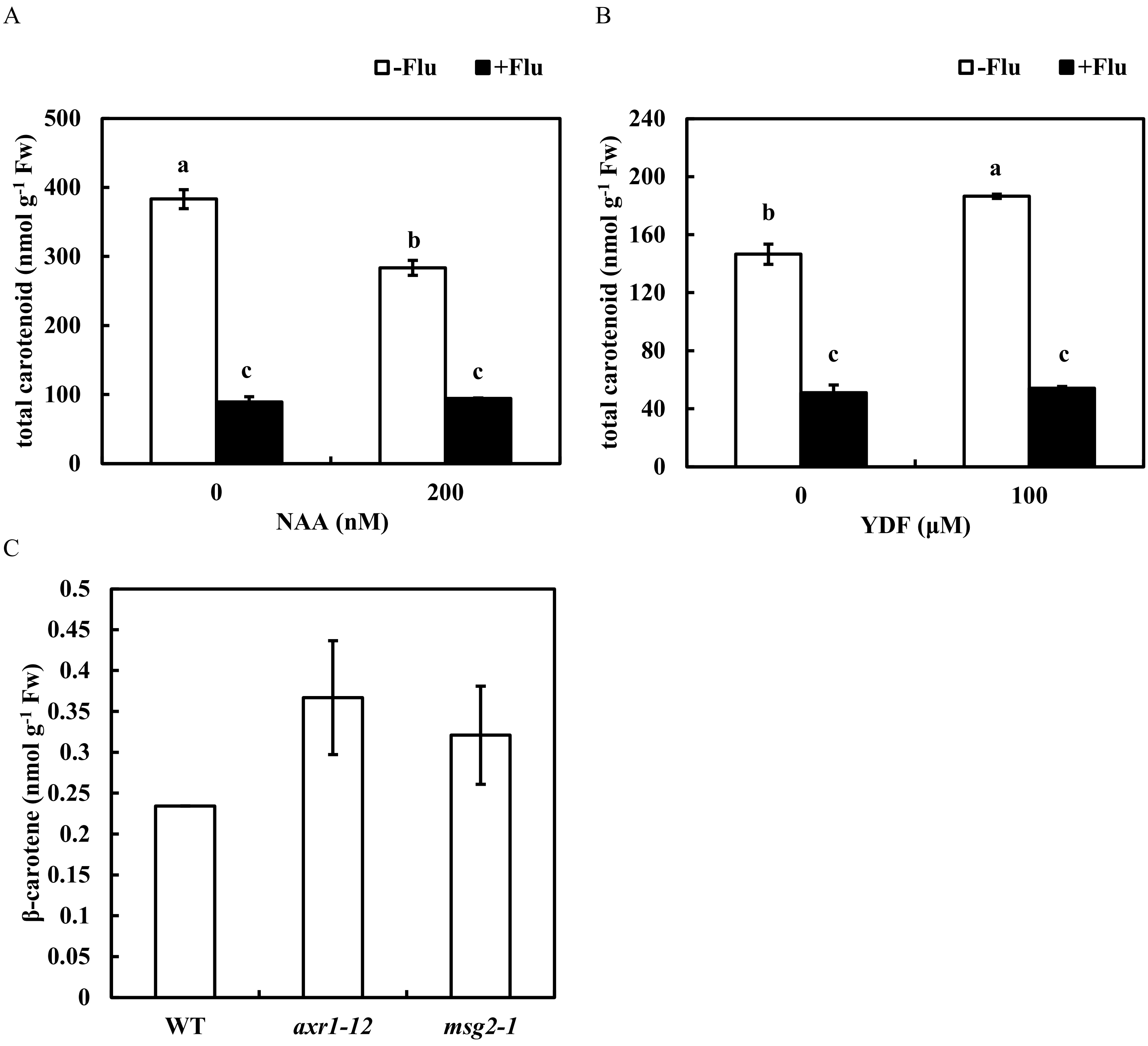


Supplementary Figure S10. Carotenoid contents in the shoots are negatively regulated by auxin, and auxin signaling mutants insignificantly increase carotenoid contents in the roots. A, B) Five-day-old plants were transferred to medium with or without 800 nM fluridone and 200 nM NAA (A) or 100 μM YDF (B). The levels of total carotenoids in the shoots were measured at 4 d after transfer (dat) in (A) and at 5 dat in (B). C) The β-carotene levels of the auxin signaling mutants in the roots were measured at 7 d after germination. Data represent the means ± SE from 3 biological replicates. Different lowercase letters above the bars in (A, B) indicate significant differences at $P < 0.05$ (one-way ANOVA following Tukey-Kramer test). No significant difference was detected between non retinal-treated and retinal-treated plants in (C) (one-way ANOVA following Tukey-Kramer test).

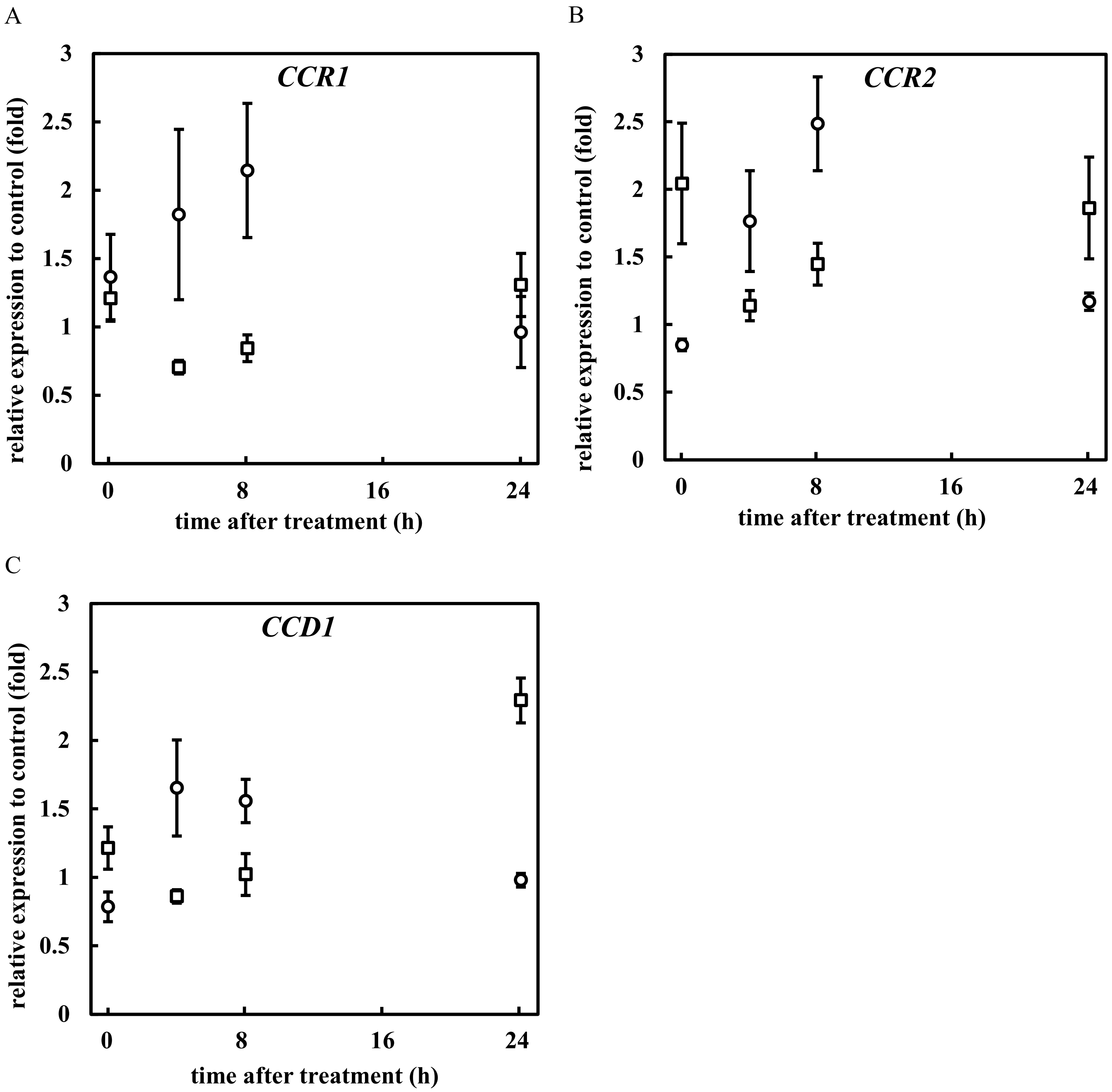


Supplementary Figure S11. The expression of *CCR1*, *CCR2* and *CCD1* is somewhat induced by fluridone. Five-day-old plants of wild-type (WT) and *mPDS* were transferred to medium with or without 800 nM fluridone at time 0. A–C) The relative expression of *CCR1*, *CCR2*, and *CCD1* to mock-treated plants after fluridone treatment in WT (open circle) and *mPDS* (open square). Data represent the means ± SE from 3 biological replicates.

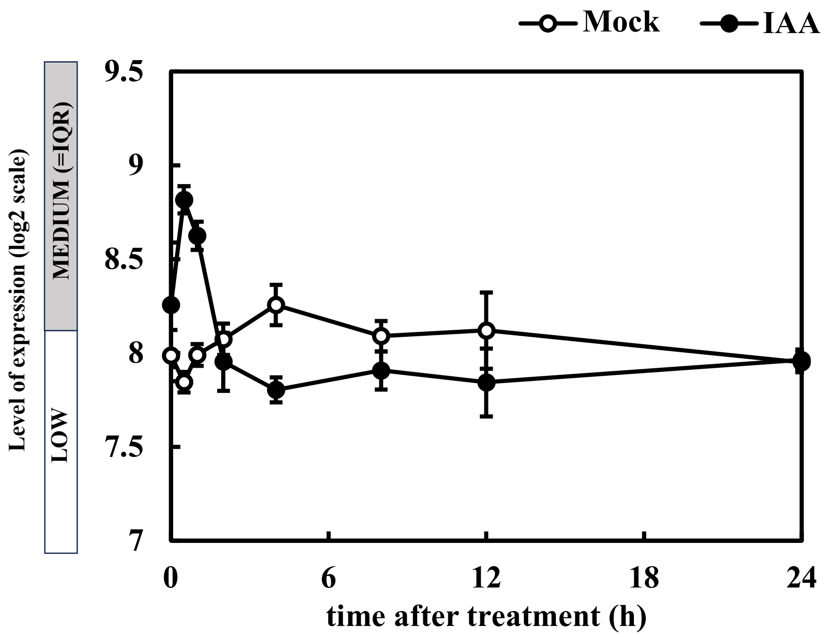


Supplementary Figure S12. *CCD7* expression is rapidly induced by exogenous IAA treatment. Public microarray data were retrieved and analyzed using GENEVESTIGATOR and the expression of *CCD7* was plotted based on Affymetrix Arabidopsis ATH1 Genome Array data (Experiment ID: AT-00655). Data are expressed as log2 scale and represent the means ± SE from 3 biological replicates.

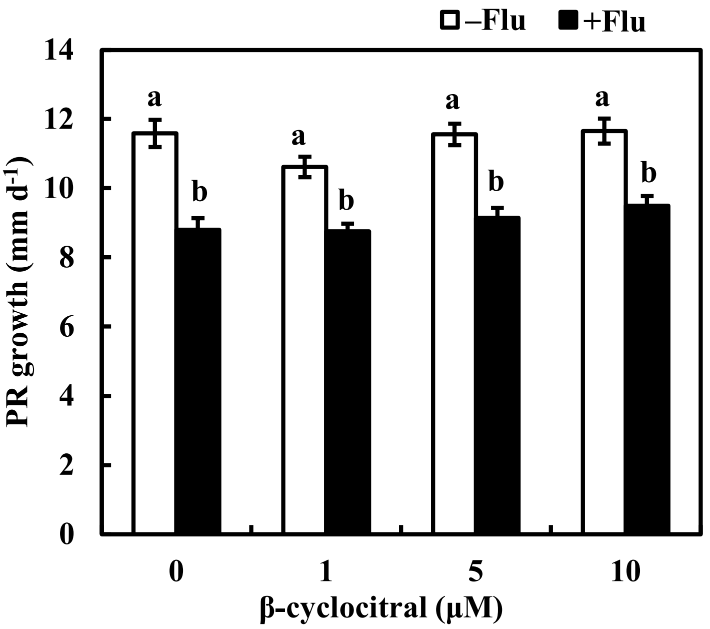


Supplementary Figure S13. β-cyclocitral is not involved in carotenoid-mediated regulation of PR growth. Five-day-old plants were transferred to medium with or without 800 nM fluridone and β-cyclocitral. The primary root (PR) growth rate was analyzed from 1 to 2 d after transfer (dat). Data represent the means ± SE from 8 seedlings. Different lowercase letters above the bars indicate significant differences at $P < 0.05$ (one-way ANOVA following Tukey-Kramer test).

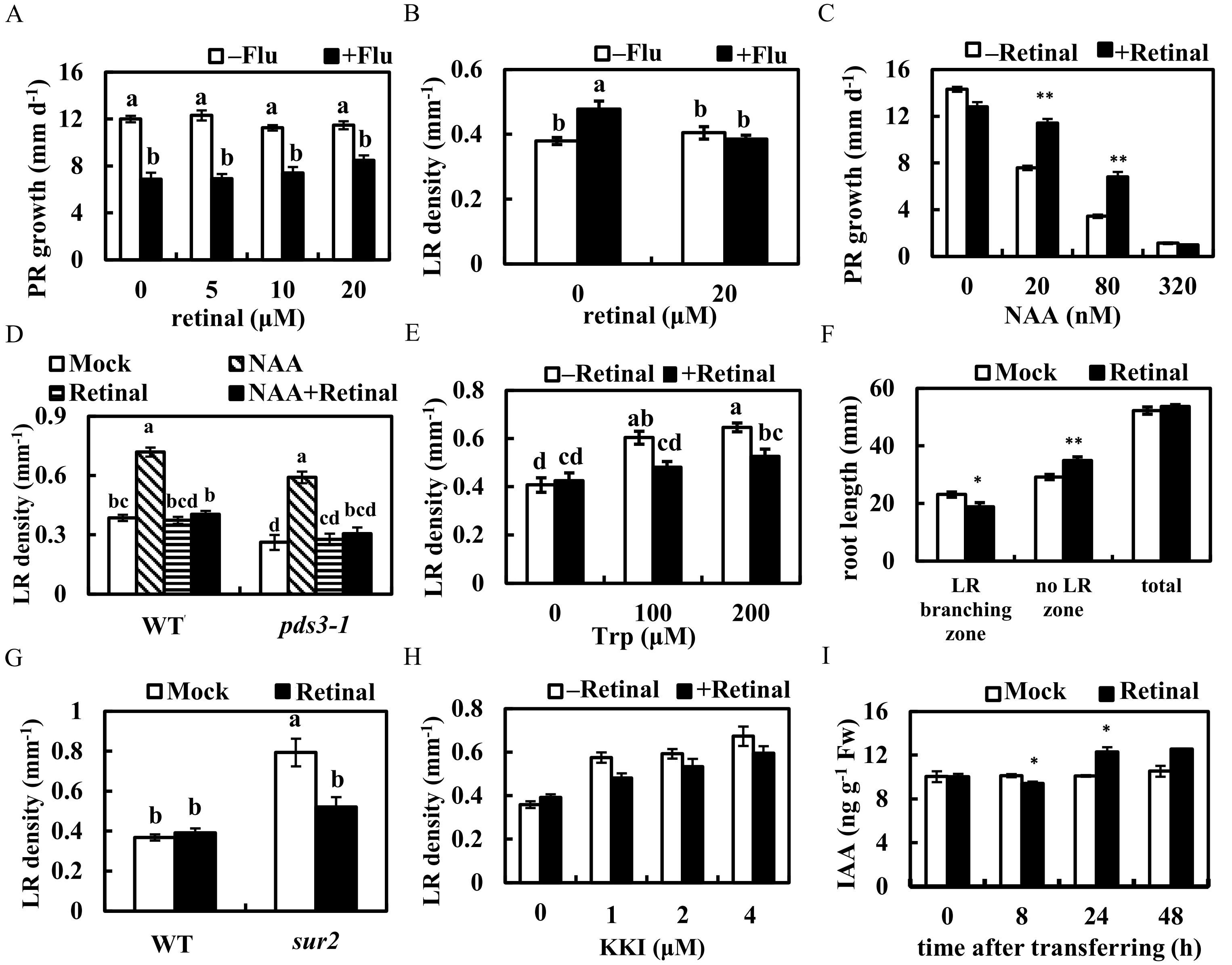


Supplementary Figure S14. Retinal is responsible for the fluridone effect and regulates auxin levels and response. A, B) Five-day-old plants were transferred to medium with or without 800 nM fluridone and retinal. C) Five-day-old plants were transferred to medium with or without 20 µM retinal and NAA. D) Five-day-old plants of wild-type (WT) and *pds3-1* were transferred to medium with or without 20 µM retinal and 20 nM NAA. E) Five-day-old plants were transferred to medium with or without 20 µM retinal and tryptophan. F) The length of lateral root (LR) branching zones (from the root–shoot junctions to the youngest LRs toward the root tips), no LR zones (from the youngest LRs to the root tips) and total roots. Five-day-old plants were transferred to medium with or without 20 µM retinal. G) Five-day-old plants of WT and *sur2* were transferred to medium with or without 20 µM retinal. H) Five-day-old plants were transferred to medium with or without 20 µM retinal and KKI. The primary root (PR) growth rate in (A, C) was measured from 2 to 3 d after transfer (dat). The root length in (F) was measured at 3 dat. The LR density in (B, D, E, G, H) was counted at 3 dat. The bars in (D) indicate mock (white), 20 nM NAA (diagonal stripe black), 20 µM retinal (horizontal striped black) and NAA + retinal (black). I) IAA quantification by retinal treatment. Five-day-old plants were transferred to medium with or without 20 µM retinal at time 0. The IAA contents in the roots were measured at the indicated time points. Data represent the means ± SE from 8 to 10 seedlings in (A–H) and from 3 biological replicates in (I). Different lowercase letters above the bars (A, B, D, E, G) indicate significant differences at $P < 0.05$ (one-way ANOVA following Tukey-Kramer test). *Significant differences compared to non retinal-treated plants in (C, F, I) (*: $P < 0.05$, **: $P < 0.01$; Student's *t*-test). No significant difference was detected between non retinal-treated and retinal-treated plants in (H) (Student's *t*-test).

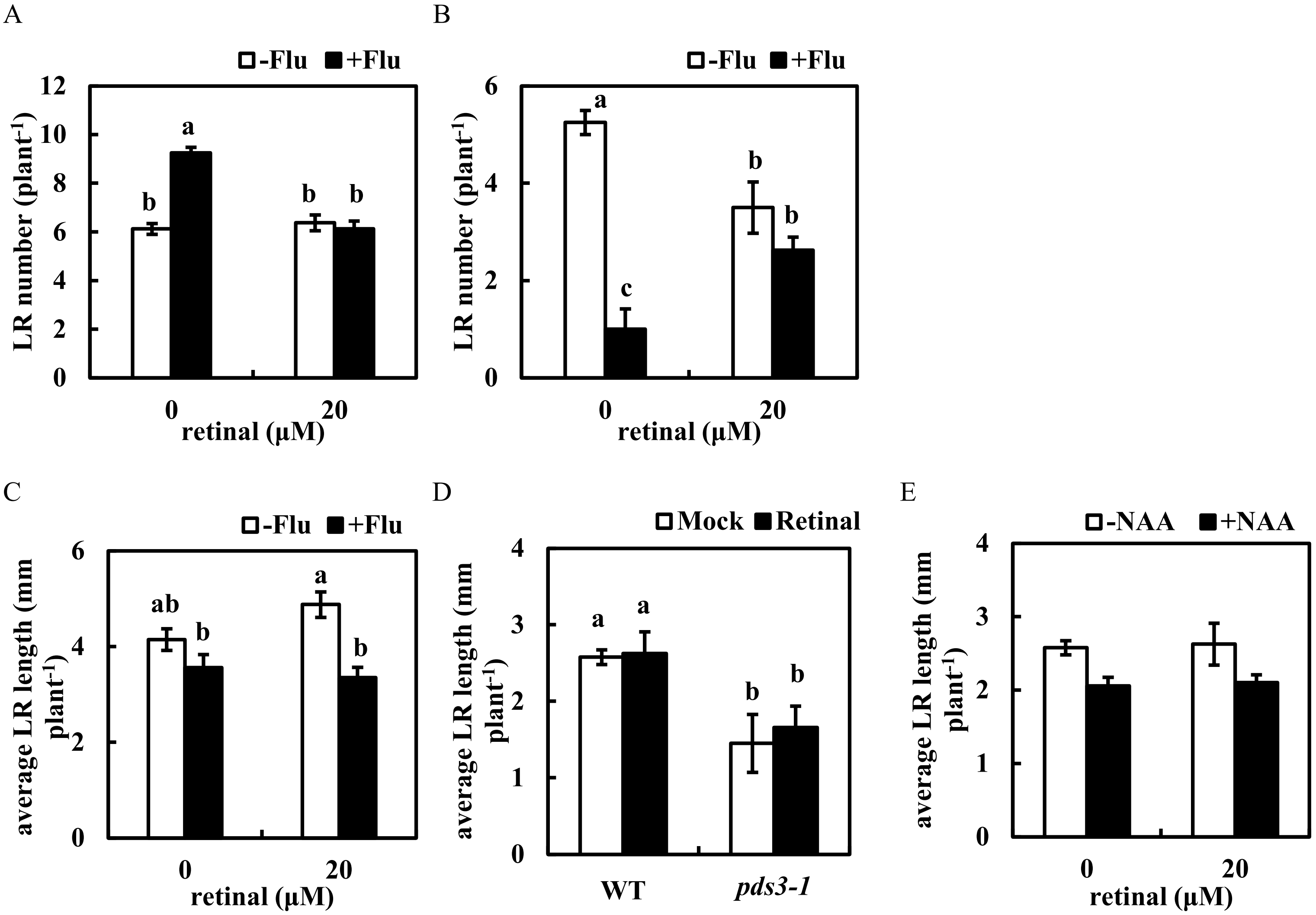


Supplementary Figure S15. Retinal restores fluridone effects on LR formation in both pre-grown and newly grown root areas but not for LR elongation. A, B) Five-day-old plants were transferred to medium with or without 800 nM fluridone and 20 μM retinal. The lateral root (LR) number in pre-grown root areas (A) and newly grown root areas (B) was counted at 4 d after transfer (dat). C, D) Five-day-old plants of wild-type (WT) and *pds3-1* were transferred to medium with or without 800 nM fluridone or 20 μM retinal. E) Five-day-old plants were transferred to medium with or without 20 nM NAA and 20 μM retinal. The average LR length was measured at 4 dat. Data represent the means ± SE from 8 seedlings in (A–C) and from 10 seedlings in (D, E). Different lowercase letters above the bars in (A–D) indicate significant differences at $P < 0.05$ (one-way ANOVA following Tukey-Kramer test). No significant difference was detected between mock-treated, retinal-treated, NAA-treated and retinal and NAA-cotreated plants in (E) (one-way ANOVA following Tukey-Kramer test).

```
A. thaliana   1 MSSSVAVLWVATSSLNPDPMNNCGLVRVLESSRLFSPCQNQRLNKGKKKQIPTWSSSFVR 60
T. aestivum   1 MATTVTLLLGAASS--PGPAAGDGAAR--------DGFQCSRLLPKKKQQRPRWVLCSLK 50
                *   *  *  * **  * *    *  *           *  **   ** * * *

A. thaliana  61 NRSRRI------------GVVSSSLVASPSGEI---ALSSEEKVYNVVLKQAALVNKQLR 105
T. aestivum  51 YGCLGVGEPGEAGGRSAASPVYSSLTVSPGGDAAVAVVSSEQKVYDVVVKQAALLKRQLR 110
                                    * ***  ** *       *** *** ** *****   ***

A. thaliana 106 SSSYDLDVKKPQDVVLPGSLSLLGEAYDRCGEVCAEYAKTFYLGTLLMTPERRKAIWAIY 165
T. aestivum 111 PSQQQQQAPPAVARELDAPRGGLGEAYARCGEICEEYAKTFYLGTLLMTEERRRAIWAIY 170
                 *             *      ***** **** * ************** *** ******

A. thaliana 166 VWCRRTDELVDGPNASHITPMALDRWEARLEDLFRGRPFDMLDAALADTVARYPVDIQPF 225
T. aestivum 171 VWCRRTDELVDGPNASHITPQALDRWERRLEDLFAGRPYDMLDAALSDTITKFPIDIQPF 230
                ******************** ****** ****** *** ******* **    * *****

A. thaliana 226 RDMIEGMRMDLKKSRYQNFDDLYLYCYYVAGTVGLMSVPVMGIDPKSKATTESVYNAALA 285
T. aestivum 231 KDMIDGMRTDLKKARYKNFDELYMYCYYVAGTVGLMSVPVMGIAPDSKATAESVYGAALA 290
                 *** *** **** ** *** ** ******************* * **** **** ****

A. thaliana 286 LGIANQLTNILRDVGEDARRGRVYLPQDELAQAGLSDEDIFAGKVTDKWRNFMKMQLKRA 345
T. aestivum 291 LGLANQLTNILRDVGEDARRGRIYLPQDELAEAGLSDEDIFKGVVTDKWRKFMKRQIKRA 350
                ** ******************* ******** ********* * ****** *** * ***

A. thaliana 346 RMFFDEAEKGVTELSAASRWPVWASLLLYRRILDEIEANDYNNFTKRAYVGKVKKIAALP 405
T. aestivum 351 RMFFEEAERGVTELRKESRWPVWASLLLYRQILDEIEANDYNNFTKRAYVGKAKKVLALP 410
                **** *** *****   ************* ********************* **  ***

A. thaliana 406 LAYAKSVLKTSSSRLSI- 422
T. aestivum 411 VAYGRSLLLPYSLRNNQT 428
                 **  * *   * *
```

```
         WT    421  GAATATGCTAAGACGTTTTATCTTGGAACTTTGCTTATGACACCCGAAAGGCGAAAGGCG  480
                    ||||||||||||||||||||||||||||||||||||||||||||||||||||||||||||
SALK_054288    421  GAATATGCTAAGACGTTTTATCTTGGAACTTTGCTTATGACACCCGAAAGGCGAAAGGCG  480
               141   E  Y  A  K  T  F  Y  L  G  T  L  L  M  T  P  E  R  R  K  A   160

         WT    481  ATTTGGGCAATCTACGTTTGGTGTAGAA...
                    ||||||||||||||||
SALK_054288    481  ATTTGGGCAATCTACG  -  T-DNA insertion sequence
               161   I  W  A  I  Y  V  W  C  R
```

Supplementary Figure S16. The alignment between *A. thaliana* PSY and *T. aestivum* PSY1 protein and the partial alignment between WT and SALK_054288 (*psy*) mutant at the PSY locus (AT5G17230). Protein alignment is performed by CLUSTALW (https://www.genome.jp/tools-bin/clustalw). Number indicates amino acid or nucleotide positions along the PSY protein or *PSY* gene. The amino acids below correspond to the WT PSY protein. Asterisks above indicate identical amino acids. Black lines below indicate identical bases. Blue lines indicate the conserved sequence for the active site lid (i.e., YAKTF, RAYV). Black frames indicate amino acids consisting the substrate binding pocket. Red frames indicate amino acids constituting the catalytic activity.

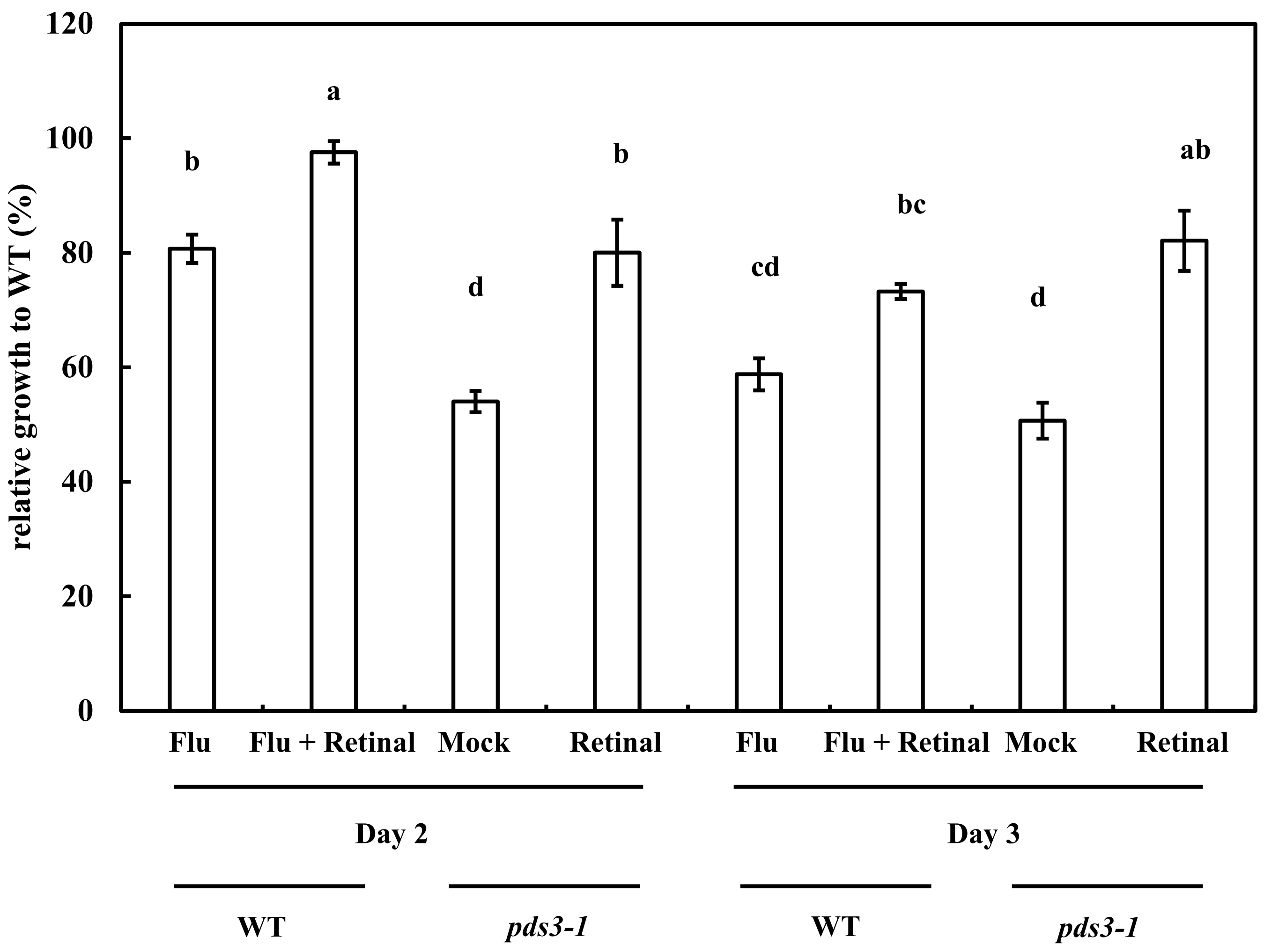


Supplementary Figure S17. The different effect of retinal to fluridone-treated WT plants and *pds3-1* plants in root growth recovery. Five-day-old plants of wild-type (WT) were transferred to medium in mock treatment, 800 nM fluridone treatment and co-treatment of 800 nM fluridone and 20 μM retinal. Five-day-old plants of *pds3-1* were transferred to medium with or without 20 μM retinal. The relative PR growth rate compared to the mock-treated WT plants was analyzed from 1 to 2 d after transfer (dat) or from 2 to 3 dat. Data represents the means ± SE from three independent technical replicates. Different lowercase letters above the bars indicate significant differences at $P < 0.05$ (one-way ANOVA following Tukey-Kramer test).

Supplementary Table S1. Endogenous IAA contents in the shoots following fluridone treatment. Five-day-old plants were transferred to mock or 800 nM fluridone medium at time 0. The IAA contents in the shoots were measured at indicated timepoints. The results were indicated by mean ± SE (n = 3). The unit is ng $g^{-1}$ Fw.

| | 0 h | 8 h | 24 h |
|---|---|---|---|
| Control | 11.41 ± 1.63 | 12.92 ± 1.40 | 11.55 ± 1.25 |
| Fluridone | 11.35 ± 1.23 | 11.44 ± 1.01 | 10.86 ± 0.84 |

Supplementary Table S2. Gene-specific primers used for RT–qPCR analysis.

| Gene name | Primer name | Primer Sequence (5'-3') | Reference |
|---|---|---|---|
| *ACTIN2* | ACTIN2-27S | 5'-CGCTCTTTCTTTCCAAGCTCATA-3' | Xu et al., 2017 |
| | ACTIN2+55 AS | 5'-CCATACCGGTACCATTGTCACA-3' | |
| *IAA19* | IAA19+390S | 5'-CTTCGGTTTCCGTGGCATCG-3' | Xu et al., 2017 |
| | IAA19+521 AS | 5'-CATGACTCTAGAAACATCCC-3' | |
| *YUC9* | YUC9+542S | 5'-ATAAGTCCGGCGAGAAATTCAGAG-3' | Xu et al., 2017 |
| | YUC9+682 AS | 5'-TCGGTAAAACATGAACCGAG-3' | |
| *CCR1* | CCR1+3599 S | 5'-AAGAGCAAGCGTTTGACAGGTAAG-3' | This study |
| | CCR1+3678 AS | 5'-TACGATTGCGATGAAGGAACTGG-3' | |
| *CCR2* | CCR2+2888 S | 5'-CATCGGGTAGCTGCTGATATTGGG-3' | This study |
| | CCR2+2968 AS | 5'-AGCCAACCAAGTAAACCAAGAAGG-3' | |
| *CCD1* | CCD1+2574 S | 5'-TATGTTCCGCGTGAGACAGCAG-3' | This study |
| | CCD1+2665 AS | 5'-TCACAGTCACGCATGATTTCCC-3 | |
| *CCD7* | CCD7+2077 S | 5'-CGTTGGTGAGCCCATGTTTGTC-3' | This study |
| | CCD7+2180 AS | 5'-TCTCTCCACCGAAACCGCATACTC-3' | |
| *CCD8* | CCD8+2715 S | 5'-GTGCAACCCATGAGGATGATGGAG-3' | This study |
| | CCD8+2839 AS | 5'-CCATAGGGAAACTTGGCTCTTGC-3' | |

**Xu D, Miao J, Yumoto E, Yokota T, Asahina M, Watahiki M** (2017) *YUCCA9*-mediated auxin biosynthesis and polar auxin transport synergistically regulate regeneration of root systems following root cutting. *Plant and Cell Physiology* **58:** 1710-1723

Supplementary Table S3. PR growth rate in auxin-related mutants by mock treatment. The results were indicated by the mean ± SE (n = 20 for signaling and polar transport mutants, n = 8 for the other mutants).

| Genotype | Growth rate (mm $d^{-1}$) |
|---|---|
| WT (For signaling and polar transport mutants) | 15.40 ± 0.19 |
| *msg2-1* | 16.34 ± 0.23 |
| *slr-1* | 11.74 ± 0.38 |
| *arf19-1i/nph4-1* | 12.45 ± 0.37 |
| *axr1-12* | 17.30 ± 0.33 |
| *aux1-21* | 9.30 ± 0.83 |
| | |
| WT (For *wei2*, *wei7* and *wei2 wei7* mutants) | 9.89 ± 0.42 |
| *wei2-1* | 9.99 ± 0.49 |
| *wei7-1* | 10.25 ± 0.16 |
| *wei2-1 wei7-1* | 1.60 ± 0.19 |
| | |
| WT (For *yuc* mutants) | 12.18 ± 0.34 |
| *yuc1* | 12.33 ± 0.40 |
| *yuc2* | 11.94 ± 0.25 |
| *yuc3* | 11.49 ± 0.63 |
| *yuc4* | 11.98 ± 0.63 |
| *yuc6* | 11.21 ± 0.84 |
| *yuc7* | 11.43 ± 0.50 |
| *yuc8* | 12.64 ± 0.26 |
| *yuc9* | 11.04 ± 0.55 |
| *yuc10* | 8.99 ± 0.69 |
| *yuc11* | 11.44 ± 0.50 |
| *yucQ* | 3.83 ± 0.53 |
| | |
| Ler (For *yuc5* mutant) | 10.89 ± 0.20 |
| *yuc5* | 8.93 ± 0.27 |
