# Supplementary material for: Exogenous application of the apocarotenoid retinaldehyde negatively regulates auxin-mediated root growth
Source: Plant Physiol. 2024 Aug 9;196(2):1659–73. doi: 10.1093/plphys/kiae405 (PMC11483604; doi:10.1093/plphys/kiae405)
Supplement: kiae405_Supplementary_Data [file pp.00788.2024-s07.pdf › pp.00788.2024-s07.pdf]

Plant Physiology®

# The exogenous application of the apocarotenoid retinaldehyde negatively regulates auxin-mediated root growth

Kang Xu, Haoran Zeng, Feiyang Lin, Emi Yumoto, Masashi Asahina, Ken-ichiro Hayashi, Hidehiro Fukaki, Hisashi Ito, and Masaaki Watahiki

**Review Timeline:**

| | |
|---|---|
| Submission Date: | 29-Aug-2023 |
| Editorial Decision: | 03-Oct-2023 |
| Resubmission Received: | 21-May-2024 |
| Editorial Decision: | 17-Jun-2024 |
| Revision Received: | 02-Jul-2024 |
| Accepted: | 03-Jul-2024 |

03-Oct-2023

Prof. Dr. Masaaki K. Watahiki
Hokkaido University
Sapporo
Japan

RE: Carotenoid metabolism negatively regulates auxin-mediated root growth

Dear Watahiki san:

Thank you for giving us the opportunity to review your manuscript for publication in the Plant Physiology. The reviewers have found the manuscript of interest, but have raised a number of concerns that will require your attention. As these concerns are of some gravity and will entail substantial changes to the manuscript, you are likely to need additional time to address them. I must therefore reject the manuscript in its present form. Nonetheless, if you are able to address the points raised in review, I would welcome your resubmission of a manuscript that incorporates your responses.

In particular, I direct your attention to the following comments for consideration in a resubmitted manuscript.

Reviewer #1 is pointing out the discrepancy between the phenotypes induced by fluridone in this study and by inhibition of carotenoid biosynthesis in previous studies. Both Reviewer #1 and Reviewer #2 are asking for data on apocarotenoid hormones (ABA, strigolactone). Further, Reveiwer #2 is concerned about the significance of minimal differences found in some of the experiments.

Please note that any resubmission will be treated as a new entry to the system and is likely to go out for review.

If you do have any queries or concerns, you are welcome to contact me in the first instance.

Thank you again for allowing Plant Physiology to review your work. We look forward to hearing from you soon.

Sincerely,

Editor, Plant Physiology

--------------------------------------------------------------------------- Reviewer comments:
Reviewer #1 (Comments for the Author):

Overall, this is an exciting paper that shows an interaction with auxin and retinal in root development. Retinal is a fairly novel regulatory compound, and this the first paper, to my knowledge that starts to unravel the relationship between auxin and retinal. The manuscript proposes a relationship between carotenoid levels and auxin - in particular, that inhibition of the carotenoid pathway increases auxin. Furthermore, auxin-deficient mutants were shown to increase beta-carotene, a major carotenoid. Retinal, a derivative of beta-carotene, inhibited the phenotypic effects of auxin treatment as well as an auxin inducible reporter. These relationships are novel and potentially important. However, there is a major concern with this report, which is that the main compound used to alter the carotenoid pathway (an inhibitor called fluridone) does not confer the expected phenotypes, based on previous literature. As this compound is used for a number of key experiments throughout the paper, I have several major concerns about the interpretation of these results.

Major Comments:
1) The fluridone data from this paper does not match previously published phenotypes (A brief list of examples: DOI:10.1093/jxb/erq186, https://doi.org/10.1093/mp/sst020, https://doi.org/10.1111/j.1365-313X.2010.04454.x). In particular, previous work on norflurazon and carotenoid mutants (for instance, the Van Norman PNAS 2014 paper that looks at lateral root development in Arabidopsis - https://doi.org/10.1073/pnas.1403016111) has shown that inhibition of carotenoid biosynthesis produces albino plants with dramatically reduced lateral root formation and reduction of DR5:LUC expression. Here, the authors show a level of fluridone treatment that reduces chlorophyll levels (although the plants still have green leaves), but increases lateral root density and increases DR5:LUC levels. The norflurazon treatments in this manuscript appear to have the same phenotype, which is very different than what has been previously published. The major concern is understanding why there is such a strong discrepancy between this new data and data that has been previously reported. It may be that fluridone is not changing carotenoid and apocarotenoid levels in the way that is expected, or there may be other reasons for the discrepancies (see my last subpoint). These are crucial questions that need to be addressed. In particular, I suggest the following:
a. Carotenoid and apocarotenoid levels should be quantified in fluridone treatment. (Total carotenoids are measured, but

individual carotenoids are not shown). The authors propose that retinal levels are decreased in fluridone treatment, but there is no evidence for this. Retinal has been shown to be critical for lateral root development, but the fluridone results from this paper suggests that decreasing retinal increases lateral root development. So it's very important to know whether fluridone is actually changing retinal levels, and if so, how much.
b. The retinal/carotenoid inhibition results should also be repeated with the psy mutant (or a pds mutant) that has the previously described phenotype of albino leaves and few/no lateral roots.
c. Statements like those made in line 49 should be more carefully considered: "Inhibition of beta-carotene biosynthesis with the phytoene desaturase (PDS) inhibitor fluridone suppresses root growth, consequently inducing lateral root growth." This implies that reduction of root growth always induces lateral root growth, which is not true. It also ignores literature that has shown that reducing beta-carotene results in a reduction of lateral root capacity.
d. Statements like those made on line 154 should be confirmed through methods that ideally don't involve inhibitor treatments. "In this study, we found that carotenoid biosynthesis inhibition resulted in auxin level elevation and response induction in the root." This is an important and exciting conclusion, and should be validated using a mutant in carotenoid biosynthesis.
e. Any discrepancies with previously published results should be detailed in the manuscript. Possible reasons for these discrepancies should be discussed. I think a major reason for the differences may be when the fluridone was applied - here it is applied after four days of growth. Four days is enough to accumulate carotenoids/apocarotenoids and develop a number of lateral roots. Therefore, the density of lateral roots may increase, but the root tip has actually lost the capability to produce lateral roots. To address this, the authors could measure lateral root production (and related phenotypes) before and after fluridone treatment.
2) Reducing beta-carotene could also affect the major apocarotenoid hormones strigolactone and ABA. The authors examine strigolactone, but do not show the data. This should be shown. ABA is not examined at all. There are widely available mutants in the ABA pathway and even genetic reporters that would be useful to examine.

Minor Comments:
Title: Fairly unspecific. A better title would be: "Retinaldehyde negatively regulates auxin-mediated root growth."
The statement made in line 135 is not accompanied by a citation ("In addition to their role in root architecture, the apocarotenoids ionone derivatives repel or attract plant herbivores."
The carotenoid pathway is often described without a "the" at the beginning. For example, line 157: "suggesting an intermediate position of retinal in carotenoid and auxin interaction pathway." It should have a "the" before carotenoid ("suggesting an intermediate position of retinal in the carotenoid and auxin interaction pathway).
Figure 1C, Line 170: "The 35S::mHvPDS plants exhibited less bleached leaves (Fig. 1C) and less reduced carotenoid content." The "bleached leaves" phenotype was not quantified. In addition, "less reduced" is a confusing way to describe the data. Recommend saying "less of a reduction of carotenoid content upon fluridone treatment."

Reviewer #2 (Comments for the Author):

The manuscript by Xu et al., addresses the molecular mechanism underneath the primary root growth inhibition that results from the block carotenoid biosynthesis with treatments with fluridone. Fluridone is well known inhibitor of the carotenoid biosynthesis affecting the PDS enzymatic activity that results in carotenoid deficiency and the decrease in ABA and strigolactones hormones and as well as other bioactive signaling molecules derived from the carotenoid pathway. As it is well established that auxin play a major role in root growth, the authors analyze the relation of the fluridone-mediated PR inhibition to the auxin levels in these roots. Based on the analyses of different signaling and biosynthetic mutants and inhibitors of the auxin pathway, the authors showed a correlation of the accumulation of auxin and the fluridone-mediated PR inhibition. These results led them to conclude that an auxin accumulation is the primary cause of the defect in the PR growth. They also provide evidences that carotenoid accumulation and the expression of some genes related to their biosynthesis and catabolism to apocatotenoids synthesis are regulated by auxin. Finally the partial suppression of the root inhibition caused by fluridone by two apocarotenoids derived from ß-carotene (ß cyclocitral and retinal) allow to support that retinal inhibits auxin accumulation and suppress this phenotype.
In the past years it has become clear the central function of diverse carotenoid-derived molecules as retrograde signals that regulate the expression of nuclear genes modulating developmental and environmental responses in plants. However, both the mechanisms of action how these signals and the processes that regulate remain still not fully understood. In particular those related with the function of retinal. Also, it is clear that complex interactions between these plastid-derived signals and the major plant hormones to regulate diverse developmental processes. Thus, the topic addressed in the present manuscript is relevant. I think that the authors provide a good correlation that increase in the auxin levels is important for the fluridone -mediated PR inhibition. However, the correlation of a direct role of carotenoid in this response will need additional analysis. The observations are interesting but in the opinion of this reviewer several of the major conclusions are supported by relative minor differences based on few individuals. Also, the manuscript presentation will benefit of rewriting. Also including additional references of previous literature relevant for the work (ABA) and providing a more comprehensive discussion.
Here are some of the major concerns:

- Line 205. The authors conclude that fluridone-mediated PR inhibition is the result of the accumulation of auxin levels.

However, previous reports in maize have also proposed a role of ABA in the defects of root growth associated to defects in carotenoid pathway or fluridone (Saab, et al., 1990). Also a role of ABA has been found in the promotion of meristem maintenance (Ahang et al. 2010). Since fluridone treatment alters ABA levels is important that the authors take in account these findings and discuss this aspect. How the proposed ABA PR regulation fits with the findings described in this work? Perhaps it would be interesting to analyze genes that participate in the ABA .biosynthesis, particularly since this hormone is also an apocarotenoid whose levels will change by the fluridone treatment.
- Retinal has been shown to be important in lateral root formation (Dickinson 2021). Here the authors observed an increase in the LR aspect with fluridone, how the authors reconcile this with the propose participation of retinal in the fluridone response?
- One aspect that needs to be improved is the sample number used in several of the analyses that in many cases is low (5-8 plants). Increasing the sample number will probably help to further substantiate the results and support major conclusions.
- The results from Figure 1 related to the impact of fluridone in carotenoid content have been previously reported and perhaps can be moved to supplementary Figures.
- Line 200. Based on the DR5-LUCmarker how the authors conclude that fluridone activates auxin signaling?
- Using DR5-LUCmarker the authors conclude that auxin level is higher in the root tip in the fluridone-treated plants at 14h, but based on the Fig. 3 the difference at this time is minimal. Is this difference statistically significant?
- To substantiate the participation of auxin the authors analyzed different auxin signaling and biosynthetic mutants. In several cases many of these mutants affect genes that belong to families with partial redundant function. The results obtained from these analyses is variable respect to the resistance to the fluridone treatment and in many cases the observed differences are not significative. In my opinion the description of the data in section could be improved to facilitate the reader interpretation. At the end of the section (line 249) the conclusion is unclear: "Taken together, auxin biosynthesis, signaling, and catabolism may be required for carotenoid-mediated PR growth regulation".
- The authors analyze the expression of some carotenoid genes. It results intriguing to this reviewer how the authors decided to analyze the expression of some particular carotenoid biosynthetic and degradation genes. At the end they observe an induction of the CCD7 and CCD8 genes that are involved in strigalactone biosynthesis. However, based on the analysis of strigolactone biosynthetic mutants the authors do not support that this signal participate in the root responses. Thus, is not clear how this data fits into this response. Also CCR2 is not a biosynthesis gene and this needs to be clarified.
- Along the manuscript the authors have conclusions that are not supported by their data. For example in Line 265 is concluded that "endogenous auxin levels and signaling negatively control β-carotene levels" but in the same paragraph they state that the signaling mutants do not have a significant difference. These type of statements need to be corrected along the manuscript.
- The authors show that the addition of retinal (20µM) partially suppress the fluridone-root inhibition. Previous reports have demonstrated that 1µM of retinal is sufficient to restore the lateral root retinoid-mediated role. Although is possible that different responses require different level of a signal, the level of retinal use here is 20X higher and it would be important that the authors comment whether this concentration would not expect to result in potential artefacts. This aspect merits some comment or proper reference.
- The Discussion is not easy to follow and will also benefit from rewriting, avoiding to be a summary of the results and more the interpretation and discussion of them in respect to what is known in the field.
- Perhaps of the authors include in their model the two conditions (with and without) fluridone or growth will be useful.

Minor points
Figure 7C. I am confused with this panel. as in theory is a comparison - and + retinal.
Figure legends need to be more specific including the sample size and the exact.

Dear Dr.reviewers

On behalf of all authors of the manuscript, (PP2023-RA-01324): " Carotenoid metabolism negatively regulates auxin-mediated root growth", I would like to thank for valuable comments and suggestions again. Here, we answer all comments point-by-point and the manuscript is revised and updated.

After the revision, the manuscript is resubmitted with new title “The exogenous application of the apocarotenoid retinaldehyde negatively regulates auxin-mediated root growth”.

All the authors have read the revised materials and have approved this revision.

Sincerely,
Masaaki K. Watahiki

Division of Biological Sciences
Faculty of Science
Hokkaido University
Sapporo, Japan
watahiki@sci.hokudai.ac.jp

**Reviewer: 1**

**Major comments:**

**Comment-rev1-1:**

The fluridone data from this paper does not match previously published phenotypes (A brief list of examples: DOI:10.1093/jxb/erq186, https://doi.org/10.1093/mp/sst020, https://doi.org/10.1111/j.1365-313X.2010.04454.x). In particular, previous work on norflurazon and carotenoid mutants (for instance, the Van Norman PNAS 2014 paper that looks at lateral root development in Arabidopsis - https://doi.org/10.1073/pnas.1403016111) has shown that inhibition of carotenoid biosynthesis produces albino plants with dramatically reduced lateral root formation and reduction of *DR5:LUC* expression. Here, the authors show a level of fluridone treatment that reduces chlorophyll levels (although the plants still have green leaves), but increases lateral root density and increases *DR5:LUC* levels. The norflurazon treatments in this manuscript appear to have the same phenotype, which is very different than what has been previously published. The major concern is understanding why there is such a strong discrepancy between this new data and data that has been previously reported. It may be that fluridone is not changing carotenoid and apocarotenoid levels in the way that is expected, or there may be other reasons for the discrepancies (see my last subpoint). These are crucial questions that need to be addressed.

**Answer:** We thank for this comment greatly, and we understand it is quite important to describe and explain the discrepancy that occurred between this study and previous research. For the partial bleaching (only newly emerged true leaves) phenotype, we consider the reason as short-term inhibition of carotenoid biosynthesis mainly targets newly synthesized carotenoids but has less effect on the carotenoid pool. The high stability of carotenoids with sequestration in mature plastids (Merzlyak and Solovchenko, 2002; Sun et al., 2018) and the potency of PDS proteins as carotenoid transporter (Lopez et al., 2008) support this hypothesis. Application of fluridone to maize leaves causes the leaf to grow into a white/green mixed leaf: white in basal part, pale green in middle part and green in apical part (Kim et al., 2004). For root architecture, we consider there is no conflict between this study and previous research (for instance, Van Norman et al., 2014). Van Norman et al. (2014) described root architecture change under long-term carotenoid deficiency. This study focuses on a rapid response to the temporary inhibition of carotenoid biosynthesis. We discuss this issue in line 380-408.

**Kim JS, Yun BW, Choi JS, Kim TJ, Kwak SS, Cho KY** (2004) Death mechanisms caused by carotenoid

biosynthesis inhibitors in green and in undeveloped plant tissues. *Pesticide Biochemistry and Physiology* **78:** 127-139

**Lopez AB, Yang Y, Thannhauser TW, Li L** (2008) Phytoene desaturase is present in a large protein complex in the plastid membrane. *Physiologia Plantarum* **133:** 190-198

**Merzlyak MN, Solovchenko AE** (2002) Photostability of pigments in ripening apple fruit: a possible photoprotective role of carotenoids during plant senescence. *Plant Science* **163:** 881-888

**Sun T, Yuan H, Cao H, Yazdani M, Tadmor Y, Li L** (2018) Carotenoid metabolism in plants: the role of plastids. *Molecular Plant* **11:** 58-74

**Van Norman JM, Zhang J, Cazzonelli CI, Pogson BJ, Harrison PJ, Bugg TD, Chan KX, Thompson AJ, Benfey PN** (2014) Periodic root branching in *Arabidopsis* requires synthesis of an uncharacterized carotenoid derivative. *Proceedings of the National Academy of Sciences, USA* **111:** E1300-E1309

**Comment-rev1-1 (a):**

Carotenoid and apocarotenoid levels should be quantified in fluridone treatment. (Total carotenoids are measured, but individual carotenoids are not shown). The authors propose that retinal levels are decreased in fluridone treatment, but there is no evidence for this. Retinal has been shown to be critical for lateral root development, but the fluridone results from this paper suggests that decreasing retinal increases lateral root development. So it's very important to know whether fluridone is actually changing retinal levels, and if so, how much.

**Answer:** We thank for this comment. Individual graphs of chlorophyll and carotenoid content reduction by fluridone treatment are added as supplemental materials (Supplemental Fig. S4, B–G). Retinal is an apocarotenoid that is less studied in plants, and the endogenous biosynthesis pathway and function are poorly understood yet. We propose that the retinal levels are decreased by fluridone from the result that exogenous retinal application fully complemented fluridone-induced PR growth suppression and largely encouraged the PR growth of *pds3-1* mutant (Fig. 7, A and B). This study focuses on the function of exogenous retinal complementary to fluridone to investigate the short-term carotenoid

deficiency regulating root growth, so we have no plan to quantify the endogenous retinal levels in this study, but it is a consideration for future research.

**Comment-rev1-1 (b):**

The retinal/carotenoid inhibition results should also be repeated with the *psy* mutant (or a *pds* mutant) that has the previously described phenotype of albino leaves and few/no lateral roots.

**Answer:** We appreciate greatly this suggestion, thus we employed *psy* mutant (the same allele used in Van Norman's work), and *pds3-1*. *psy* and *pds3-1* exhibited an albino phenotype. *pds3-1* showed a complete resistance to fluridone on PR growth and LR density (Fig. 1, E–G). However, *psy* mutant was partially resistant to fluridone on PR growth suppression, and its LR formation was induced by fluridone (Supplemental Fig. S1, A and B). On the other hand, retinal application enhanced PR growth of *pds3-1*, but did not affect LR density (Fig. 7B; Supplemental Fig. S13D). These results indicate that fluridone-induced root architecture change is dependent on PDS activity, and retinal is responsible for PDS-mediated PR growth regulation.

**Comment-rev1-1 (c):**

Statements like those made in line 49 should be more carefully considered: "Inhibition of beta-carotene biosynthesis with the phytoene desaturase (PDS) inhibitor fluridone suppresses root growth, consequently inducing lateral root growth." This implies that reduction of root growth always induces lateral root growth, which is not true. It also ignores literature that has shown that reducing beta-carotene results in a reduction of lateral root capacity.

**Answer:** We thank for this correction. This study focuses on the mechanism of fluridone-mediated primary root growth suppression in short-term treatment. Thus the sentence is modified as "In this study, we show that short-term inhibition of PDS by fluridone suppressed PR growth in WT, but to a lesser extent in auxin mutants." (line 49-50). We carefully checked these kinds of logical mistakes throughout the manuscript.

**Comment-rev1-1 (d):**

Statements like those made on line 154 should be confirmed through methods that ideally don't involve inhibitor treatments. "In this study, we found that carotenoid biosynthesis inhibition resulted in auxin level elevation and response induction in the root." This is an

important and exciting conclusion, and should be validated using a mutant in carotenoid biosynthesis.

**Answer:** We thank for this suggestion. In this study, we focus on how the temporary PDS inhibition regulates root growth. Thus we applied fluridone to 5-day-old seedlings to observe the root phenotypes. Although it is important to prove the increase of auxin levels in carotenoid-deficient mutants, we are afraid that the mechanisms of root growth regulation of short-term and long-term carotenoid deficiency may be different, as described in line 382-408. Instead, we employed fluridone-resistant transgenic plant *35S::mHvPDS* to confirm the fluridone-mediated auxin increase (Fig. 3B; Supplemental Fig. S7B).

**Comment-rev1-1 (e):**

Any discrepancies with previously published results should be detailed in the manuscript. Possible reasons for these discrepancies should be discussed. I think a major reason for the differences may be when the fluridone was applied - here it is applied after four days of growth. Four days is enough to accumulate carotenoids/apocarotenoids and develop a number of lateral roots. Therefore, the density of lateral roots may increase, but the root tip has actually lost the capability to produce lateral roots. To address this, the authors could measure lateral root production (and related phenotypes) before and after fluridone treatment.

**Answer:** We thank for this comment. Indeed, it is a great way to identify the difference of fluridone effect on pre-grown roots and newly elongated roots. The result showed that in pre-grown root areas, fluridone slightly induced LR formation. However, in newly grown root areas, LR formation was severely arrested by fluridone treatment (Supplemental Fig. S3), referring the different response of new and old organs to fluridone treatment.

**Comment-rev1-2:**

Reducing beta-carotene could also affect the major apocarotenoid hormones strigolactone and ABA. The authors examine strigolactone, but do not show the data. This should be shown. ABA is not examined at all. There are widely available mutants in the ABA pathway and even genetic reporters that would be useful to examine.

**Answer:** We thank for this comment. The ABA- and strigolactone-related mutants were examined for fluridone resistance, and exogenous ABA or strigolactone application was examined to determine whether it could complement fluridone-induced suppression of root growth or

not. The results showed that ABA and SL mutants had fluridone resistance comparable to WT (Supplemental Fig. S6, A–F), and exogenous ABA and strigolactones could not complement the suppression of root growth induced by fluridone (Supplemental Fig. S6, G and H). These results indicate that ABA and strigolactones may not be involved in fluridone-mediated PR suppression pathway.

**Minor comments**

**Comment-rev1-1:**

Title: Fairly unspecific. A better title would be: "Retinaldehyde negatively regulates auxin-mediated root growth."

**Answer:** We appreciate this advice. With the revision of manuscript, we changed the title to “The exogenous application of the apocarotenoid retinaldehyde negatively regulates auxin-mediated root growth” to match to our focus.

**Comment-rev1-2:**

The statement made in line 135 is not accompanied by a citation ("In addition to their role in root architecture, the apocarotenoids ionone derivatives repel or attract plant herbivores."

**Answer:** We thank for pointing this mistake out. With the revision of the manuscript, we deleted the description of non-bioactive apocarotenoids to make the Introduction more focused.

**Comment-rev1-3:**

The carotenoid pathway is often described without a "the" at the beginning. For example, line 157: "suggesting an intermediate position of retinal in carotenoid and auxin interaction pathway." It should have a "the" before carotenoid ("suggesting an intermediate position of retinal in the carotenoid and auxin interaction pathway).

**Answer:** We thank for pointing this kind of mistake out. With the revision of the manuscript, we checked throughout the manuscript to add “the” in proper positions.

**Comment-rev1-4:**

Figure 1C, Line 170: "The *35S::mHvPDS* plants exhibited less bleached leaves (Fig. 1C) and less reduced carotenoid content." The "bleached leaves" phenotype was not quantified.

In addition, "less reduced" is a confusing way to describe the data. Recommend saying "less of a reduction of carotenoid content upon fluridone treatment."

**Answer:** We thank for this suggestion. The description here has been modified as "The *35S::mHvPDS* plants were confirmed to have resistance to fluridone in terms of chlorophyll and carotenoid contents" to avoid misunderstanding (line 142-143).

**Reviewer: 2**

**Major comments:**

**Comment-rev2-1:**

Line 205. The authors conclude that fluridone-mediated PR inhibition is the result of the accumulation of auxin levels. However, previous reports in maize have also proposed a role of ABA in the defects of root growth associated to defects in carotenoid pathway or fluridone (Saab, et al., 1990). Also a role of ABA has been found in the promotion of meristem maintenance (Ahang et al. 2010). Since fluridone treatment alters ABA levels is important that the authors take in account these findings and discuss this aspect. How the proposed ABA PR regulation fits with the findings described in this work? Perhaps it would be interesting to analyze genes that participate in the ABA biosynthesis, particularly since this hormone is also an apocarotenoid whose levels will change by the fluridone treatment.

**Answer:** We thank for this comment. The ABA- and strigolactone-related mutants were examined for fluridone resistance, and exogenous ABA or strigolactone application was examined to determine whether it could complement fluridone-induced suppression of root growth or not. The results showed that ABA and SL mutants had fluridone resistance comparable to WT (Supplemental Fig. S6, A–F), and exogenous ABA and strigolactones could not complement the suppression of root growth induced by fluridone (Supplemental Fig. S6, G and H). These results indicate that ABA and strigolactones may not be involved in fluridone-mediated PR suppression pathway.

**Comment-rev2-2:**

Retinal has been shown to be important in lateral root formation (Dickinson 2021). Here the authors observed an increase in the LR aspect with fluridone, how the authors reconcile this with the propose participation of retinal in the fluridone response?

**Answer:** We thank for this comment. In this paper the short-term application of fluridone induced the LR number in the pre-grown root areas but reduced it in the newly grown root areas, resulting in the increase in LR density (Fig. 1D; Supplemental Fig. S3). It indicates that the effect of fluridone on new and old organs is different. The application of retinal in this study complemented fluridone-induced suppression of root growth and induction of LR formation (Fig. 7A; Supplemental Fig. S13B), and complemented the short PRs of *pds3-1* mutant (Fig. 7B), indicating that retinal is responsible for PDS-mediated root architecture change. Although retinal did not alter LR density in WT plants, it did shift the sites of LR emergence away from the root tips and delay the LR emergence (Supplemental Fig. S13F), suggesting that retinal may reduce auxin response. It is reasonable that fluridone increases endogenous auxin levels and activates auxin response via inhibiting retinal biosynthesis. One of the possible reason for this discrepancy may refer that in Dickinson's paper they measured LR capacity by excising the root tips, which is similar to the root cut response to encourage LR formation (Xu et al., 2017).

**Comment-rev2-3:**

One aspect that needs to be improved is the sample number used in several of the analyses that in many cases is low (5-8 plants). Increasing the sample number will probably help to further substantiate the results and support major conclusions.

**Answer:** We thank for this comment. Although 5-8 plants are not very much, the procedure that transferring seedlings with similar primary root lengths could reduce the variations to a certain extent. In fact, most experiments had small standard error. However, we noticed that the experiment of fluridone-treated auxin signaling and transport mutants had large SE and resulted in ambiguous significance (Fig. 4A). So we repeated the experiment with 20 seedlings. Followed by an increased n number and reduced SE, ANOVA and Turkey-Kramer test was done. The results showed that *msg2-1*, *slr-1* and *arf7 arf19* had insignificant resistance to fluridone, while *axr1-12* and *aux1-21* was significantly resistant to fluridone (Fig. 4A).

**Comment-rev2-4:**

The results from Figure 1 related to the impact of fluridone in carotenoid content have been previously reported and perhaps can be moved to supplementary Figures.

**Answer:** We thank for this comment. We deleted quantification of total carotenoid contents in main figure, instead we added quantification of the individual carotenoid and chlorophyll

contents as Supplemental Fig. S4, B–G.

**Comment-rev2-5:**

Line 200. Based on the *DR5-LUC*marker how the authors conclude that fluridone activates auxin signaling?

**Answer:** We thank for this comment. *DR5:LUC* is considered to be an auxin response reporter. In the manuscript we showed the *DR5:LUC* expression were induced by fluridone at the root tips. Also from the movie we can see that the *DR5:LUC* expression in the upper part of root was induced by fluridone (Supplemental Movie S1). Thus we conclude that auxin response was activated by fluridone.

**Comment-rev2-6:**

Using DR5-LUC marker the authors conclude that auxin level is higher in the root tip in the fluridone-treated plants at 14h, but based on the Fig. 3 the difference at this time is minimal. Is this difference statistically significant?

**Answer:** We thank for pointing out the incorrect explanation. To improve the data quality, we performed the *DR5:LUC* expression experiment with improved design (see Materials and Methods line 495-501). In brief, seedlings were under preincubation in D-luciferin condition for 1 d before data achievement to wait for the stability of the *DR5:LUC* expression. The results showed that auxin response was induced in the root tip in fluridone-treated wild-type plants from the beginning of treatment (about 4 h) but not for *35S::mHvPDS* transgenic plants (Fig. 3) (the first 2 h *DR5:LUC* expression increase is caused by the artificial transferring which is explained in manuscript line 180-181).

**Comment-rev2-7:**

To substantiate the participation of auxin the authors analyzed different auxin signaling and biosynthetic mutants. In several cases many of these mutants affect genes that belong to families with partial redundant function. The results obtained from these analyses is variable respect to the resistance to the fluridone treatment and in many cases the observed differences are not significative. In my opinion the description of the data in section could be improved to facilitate the reader interpretation. At the end of the section (line 249) the conclusion is unclear: "Taken together, auxin biosynthesis, signaling, and catabolism may be required for carotenoid-mediated PR growth regulation".

**Answer:** We thank for this comment. Indeed, AUX/IAA and ARF proteins are the families containing 29 and 22 members, respectively, with redundant functions (Guilfoyle and Hagen, 2007; Luo et al., 2018). The gene redundancy may lead to insignificant resistance to fluridone in these mutants. Moreover, we found that the auxin insensitivity on PR growth in auxin signaling mutants examined in this study is relevant to fluridone resistance in these mutants (Fig. 4A) (line 196-204). We consider it may make readers understand this result easily.

We thank for pointing the unclear conclusion out. With the revision of manuscript, we delete this conclusion and add conclusions for each auxin mutant examination and auxin inhibitor treatments (line 204-205, 216, 223-224, 228).

**Guilfoyle TJ, Hagen G** (2007) Auxin response factors. *Current opinion in plant biology* **10:** 453-460

**Luo J, Zhou JJ, Zhang JZ** (2018) *Aux/IAA* gene family in plants: molecular structure, regulation, and function. *International journal of molecular sciences* **19:** 259

**Comment-rev2-8:**

The authors analyze the expression of some carotenoid genes. It results intriguing to this reviewer how the authors decided to analyze the expression of some particular carotenoid biosynthetic and degradation genes. At the end they observe an induction of the *CCD7* and *CCD8* genes that are involved in strigalactone biosynthesis. However, based on the analysis of strigolactone biosynthetic mutants the authors do not support that this signal participate in the root responses. Thus, is not clear how this data fits into this response. Also CCR2 is not a biosynthesis gene and this needs to be clarified.

**Answer:** We thank for this comment. We found that β-carotene content was negatively regulated by auxin (Fig. 5). So next we decided to examine auxin whether suppresses carotenoid biosynthesis or induces carotenoid catabolism. The expression of *CCD7* and *CCD8* were largely induced by fluridone, while the carotenoid biosynthesis-related genes were less upregulated by fluridone compared to *CCD7* and *CCD8*. Thus we concluded that fluridone-induced auxin elevation may feedback to induce *CCD7* and *CCD8* expression to control carotenoid accumulation.

We thank for the correction that *CCR1* is not a carotenoid biosynthesis gene, and we revised it as "carotenoid biosynthesis-related gene" (line 253-254) since it regulates the expression of *CCR2* (Cazzonelli et al., 2009).

**Cazzonelli CI, Cuttriss AJ, Cossetto SB, Pye W, Crisp P, Whelan J, Finnegan EJ, Turnbull C, Pogson BJ** (2009) Regulation of carotenoid composition and shoot branching in *Arabidopsis* by a chromatin modifying histone methyltransferase, SDG8. *Plant Cell* **21:** 39-53

**Comment-rev2-9:**

Along the manuscript the authors have conclusions that are not supported by their data. For example in Line 265 is concluded that "endogenous auxin levels and signaling negatively control β-carotene levels" but in the same paragraph they state that the signaling mutants do not have a significant difference. These type of statements need to be corrected along the manuscript.

**Answer:** We thank for this suggestion. The role of auxin signaling in carotenoid accumulation is contributed by the application of auxin antagonist auxinole (Fig. 5A). About the insignificance in *msg2-1* and *axr1-12*, it may be because of the gene redundancy in auxin signaling since both *msg2-1* and *axr1-12* have PR growth comparable to WT (Supplemental Table. S1). In fact, *msg2-1* develops similar to WT with the defects in LR formation, and *axr1-12* has longer PR length than WT with reduced LR number (Lincoln et al., 1990; Tatematsu et al., 2004). It indicates that *msg2-1* and *axr1-12* do not have strong phenotypes and the defect in auxin signaling in these mutants may be insufficient to increase β-carotene levels like the application of auxinole.

**Lincoln C, Britton JH, Estelle M** (1990) Growth and development of the *axr1* mutants of *Arabidopsis*. *The Plant Cell* **2:** 1071-1080

**Tatematsu K, Kumagai S, Muto H, Sato A, Watahiki M, Harper R, Liscum E, Yamamoto K** (2004) *MASSUGU2* encodes Aux/IAA19, an auxin-regulated protein that functions together with the transcriptional activator NPH4/ARF7 to regulate differential growth responses of hypocotyl and formation of lateral roots in *Arabidopsis thaliana*. *The Plant Cell* **16:** 379-393

**Comment-rev2-10:**

The authors show that the addition of retinal (20µM) partially suppress the fluridone-root inhibition. Previous reports have demonstrated that 1µM of retinal is sufficient to restore the lateral root retinoid-mediated role. Although is possible that different responses require

different level of a signal, the level of retinal use here is 20X higher and it would be important that the authors comment whether this concentration would not expect to result in potential artefacts. This aspect merits some comment or proper reference.

**Answer:** We thank for this comment. At first, high dose of retinal did not alter the root growth or LR density in WT (Fig. 7B; Supplemental Fig. S13D), indicating that the effect of retinal and auxin offset each other in a competitive manner. The LR emergence in retinal-treated plants seems to be delayed in retinal-treated WT plants (Supplemental Fig. S13F), suggesting that retinal may reduce auxin signaling which is required for LR emergence. The complement effect of retinal in the root growth in *pds3-1* mutant further confirms that the retinal in this study is the downstream substrate of PDS (Fig. 7B). The related comments are stated in line 354-365.

**Comment-rev2-11:**

The Discussion is not easy to follow and will also benefit from rewriting, avoiding to be a summary of the results and more the interpretation and discussion of them in respect to what is known in the field.

**Answer:** We thank for the advice greatly, and we rewrite the Discussion. The updated Discussion describes the results in an informative way and provides more crosstalk with previous research to explain the results and construct the working model.

**Comment-rev2-12:**

Perhaps of the authors include in their model the two conditions (with and without) fluridone or growth will be useful.

**Answer:** We thank for this comment greatly. We raise the working model including the regulation of root growth by short- and long-term fluridone treatments. Short-term fluridone treatment increases auxin levels via the apocarotenoid retinal. Long-term fluridone treatment, which partially mimics the *pds3-1* mutant, additionally suppresses PR growth via unknown factors (Fig. 8) (line 412-415).

**Minor comments**

**Comment-rev2-1:**

Figure 7C. I am confused with this panel. as in theory is a comparison - and + retinal.

**Answer:** We thank for this comment. With the revision, the statistical analysis using Turkey-Kramer test now is in the comparison within the whole groups except for Supplemental Fig. S2, which is in comparison within each inhibitor treatment.

**Comment-rev2-2:**

Figure legends need to be more specific including the sample size and the exact.

**Answer:** We appreciate this comment. Legends have been updated adding the n number and exact descriptions throughout the manuscript.

17-Jun-2024

Prof. Dr. Masaaki K. Watahiki
Hokkaido University
Hokkaido University
Sapporo
Japan

RE: The exogenous application of the apocarotenoid retinaldehyde negatively regulates auxin-mediated root growth

Dear Dr. Masaaki Watahiki:

We are pleased to accept your manuscript for publication in the Plant Physiology. This acceptance is contingent on revision based on the comments of our reviewers. In particular, please consider the following:

Both Reviewers are asking if retinal can rescue inhibition of LR growth (or delayed emergence) in newly growing root tissues after fluridone treatment. Please consider adding such analysis results. If you need more time to conduct this analysis, please let us know.
There are still a number of questions raised by the reviewers (especially Reviewer #2). Please address these points as good as possible in the revised version of the manuscript.
Figure 8 nicely summarizes the proposed model differentiating short- and long-term regulation of PR growth mediated by carotenoids. I think you could indicate in this figure the contribution (novelty) of this study by highlighting the predictions that were derived from your experimental results (e.g. showing the corresponding dashed lines in red or in another color) .

Please highlight all changes and include a detailed annotation to changes to the text, with line numbers, and noting your responses to the comments.

To submit your revised manuscript, click:

Link Not Available

If you cannot return the revised manuscript within 8 weeks of receipt, please let us know. Otherwise, we will assume that you have elected not to revise the manuscript and are withdrawing it.

Thank you for allowing us to review your work. We look forward to hearing from you soon.

Sincerely,

Editor, Plant Physiology

------------------------------------------------------------------------------ Reviewer comments:
Reviewer #1 (Comments for the Author):

The authors have made a number of improvements to the paper, but there are still some outstanding issues.

1) I agree with the authors response, noted here "For the partial bleaching (only newly emerged true leaves) phenotype, we consider the reason as short-term inhibition of carotenoid biosynthesis mainly targets newly synthesized carotenoids but has less effect on the carotenoid pool."
This hypothesis should be clearly explained in the manuscript itself, as it is very important to understanding the various phenotypes that the authors observe. I think this discussion should be introduced early in the paper (lines 134-135 perhaps) to make it clear that short-term fluridone treatment is expected to target newly synthesized carotenoids and apocarotenoids.

2) Given the new data the authors present, they should test whether retinal can rescue the lateral roots in the newly grown tissue post-fluridone treatment. This experiment is important, because otherwise, it seems very strange that retinal affects root growth but not lateral root development, which are both regulated by auxin, especially given the results of previous work cited in the manuscript. It would also decrease the need to measure retinal levels, which the authors say is beyond the scope of this paper.

Reviewer #2 (Comments for the Author):

This manuscript by Xu et al., titled "The exogenous application of the apocarotenoid retinaldehyde negatively regulates auxin-mediated root growth", is a revised version of a previous submission. This study investigates changes in the root architecture, specifically the inhibition of the primary (PR) growth and promotion of lateral roots (LR), in response of a transient treatment with fluoridone in Arabidopsis seedlings. The authors demonstrate that this response results from the inhibition of the carotenoid biosynthetic enzyme PDS activity by fluridone. They further show that the PDS inhibition leads to increased endogenous auxin levels by suppressing the auxin catabolism, which are necessary for these responses. Additionally, they show that these fluridone-mediated responses can be partially restored by the addition of retinal, leading to the conclusion that retinal is responsible for the changes in the root architecture by modulating auxin levels in the root. Interestingly, this response is only observed in the preexisting root tissue, whereas the new growing tissue exhibits an opposite response, with LR development being inhibited.

Overall, the findings reported are novel findings and align with various observations regarding the function of carotenoid-derivatives (apocarotenoids) in the regulation of plant development. In this revised version, the authors have addressed several points raised by the reviewers in the first revision. They have strengthened their study by including an analysis of the fluoridone-mediated transient response in some carotenoid mutants, increasing the sample numbers to improve the significance of the data and revising of the text and discussion to improve clarity. However, several results still require further explanation and experimental support. These include the following:

- A major concern raised by the two initial reviewers is the potential discrepancy between the role of retinal in LR responses observed in this study and the one reported in previously studies (Van Norman 2014 and Dickinson 2021). In the revision, the authors suggest that temporal differences between the studies-specifically, short-term inhibition versus permanent lack of carotenoids-may account for the discrepancies. They also observed that the fluoridone-induced enhancement of LR development is observed only in the preexisting root tissue, while newly form tissue exhibits opposite response. Although the authors provide new data to address the reviewers´ comments, this aspect still remains unclear in the manuscript. Previous findings indicate that retinal mediates lateral root initiation (Dickinson), but it is possible that retinal functions differentially in LR initiation versus emergence. This possibility could potentially explain some of the differences observed. However, the authors did not analyze whether the observed LR promotion results from elongation of the preexisting LR primordia or the development of new primordia. Additionally, while they report the complex response to fluridone treatment between the pre-existing and new grown root tissue, is not clear why LR roots are inhibitied in the new tissues with respect to LR primordia initiation and emergence. A more detailed analysis, including in quantification of LR primordia during the treatments and the initiation events per cell number, would be highly informative and valuable in addresing and discussing the potential differences with the published literature.

- Another aspect requested by the reviewers was evidence for the decrease of retinal levels under the analyzed conditions. The authors argue that quantifying retinal levels is inherently difficult, which is understandable. However, this remains a critical point to conclusively demonstrate the participation of this apocarotenoid in the observed response. The authors note a substantial reduction in various carotenoids 3 days after fluoridone treatment (Figure S4), which could influence the levels of diverse apocarotenoid molecules, not only retinal. While the authors provide evidence that neither ABA, strigolactones, nor -cyclocitral alone seem to contribute to the observed responses (as neither of them complement the observed phenotypes), in contrast to retinal, supporting the latter as a potential effector of these responses. Nevertheless, given the diversity of apocarotenoids produced (many of unknown structures and functions), it is important for the authors to consider these findings when drawing conclusions and for a more comprehensive discussion.

- Although is not fully clear whether the quantification of the different carotenoids was performed with the whole seedlings or only root tissue, these data indicate that the fluoridone treatment results in significant changes in the overall carotenoid pool in the plant. It is unclear why the authors conclude fluridone mainly targets newly synthesized carotenoids, as stated in the reviewer´s response letter.

- As suggested by the reviewers, the authors analyzed the LR response mediated by fluridone in psy and pds3 mutants, strengthening this study. They observed that both psy and pds3 mutants exhibit PR length and fewer LR number compared to WT, consistent with previous reports. Interestingly, the psy mutant responds to the short-term NF treatment by displaying shorter PR and more LRs, but this response is not observed in the pds3 mutant. Moreover, they also found that the addition of retinal promotes PR growth in pds3. From these results, the authors concluded that PDS activity is required for root responses. However, it remains unclear how these results led the authors to conclude that a transient block of retinal is responsible for PDS-mediated root regulation. Specifically, how do the authors explain that a carotenoid/retinal transient block mediated by PDS occurs in the psy1 mutant that lacks carotenoids due to the PSY mutation? This aspect requires further clarification. Additionally, the analyses of the DR5 content after fluoridone treatment in the psy mutant, could be very informative data.

- Although this point was not raised by the reviewers, it is important to consider the diverse reports demonstrating a significant

role of PDS3 in maintaining the plastoquinone pool and generating GUN-mediated retrograde plastid signals (Pfannschmidt et al., 2009, Aluru et al., 2009, Rosso et al., 2009). In fact, NF has been shown to alter the redox balance of the PQ pool (Brausemann et al., 2017). How do these PDS roles fit with the findings of this study? This is a relevant aspect that merits consideration throughout the manuscript, as the data in this study strongly highlight the importance of the PDS activity.

- Additionally, it has been shown that the genes affected in the pds3 and NF-treated plants are not equivalent (Fourdree 2010). For example, many genes related to gibberellins are exclusively affected in pds3, explaining the dwarf phenotype observed in this mutant. Moreover notable similarities in the expression were observed between NF-treated plants and the immutants mutant that encodes an alternative oxidase (Aluru 2009).

- Line 258. As previously noted by a reviewer, is intriguing that fluridone treatment, there is a significantly induces the expression of the CCD7 and CCD8 genes (Fig 6C and D). The authors suggest that this might be a feedback response. However, these genes encoded proteins involved in carotenoid catabolism and in the synthesis strigolactones and other apocarotenoids, possibly including retinal. A comment in this would be important. It might also be interesting to analyze whether the transient response to fluridone observed here is altered in ccd8 or ccd7 single or double mutants.

- A major piece of evidence supporting the role of retinal in fluoridone-induced root architectural changes is the observed partial reversion of the fluoridone-mediated PR growth inhibition and LR formation induction. However, it remains unclear whether the inhibition of LR in the new formed tissue is also reverted? Additionally, what is the fluridone-mediated phenotype in the psy mutant with the addition of retinal?

- As mentioned by the reviewer, the level of retinal used in this study is 20 times higher than in previous reports. The authors demonstrate that this high retinal level did not affect root growth, but the conditions used for the retinal treatment analysis need to be clearly described.

- The authors propose that the difference in root growth between fluridone-treated and pds3-1 mutant may reflect process of plastid degradation versus undifferentiated plastids (lines 396-399). Are the authors suggesting that fluridone promotes degradation of the root plastids? Do the authors have any evidence in this regard or published data that supports this statement?

- The role of PDS as a carotenoid transporter is still under debate.

In conclusion, the present manuscript includes interesting findings that link PDS activity, auxin levels and root development. The authors have addressed several aspects requested by the reviewers and included new experimental data, thereby improving the manuscript and further supporting some conclusions, particularly regarding the role of auxins in these responses. However, the connection between the PDS and changes in the carotenoid-retinal levels as the casual factor of the fluridone-mediated transient response remains week and not fully supported by the data. As mentioned in the comments, there are still important aspects that need clarification prior the main conclusions of the article are fully supported.

**The common concerns and monitoring editor's comments**

**Common concern:**

Both Reviewers are asking if retinal can rescue inhibition of LR growth (or delayed emergence) in newly growing root tissues after fluridone treatment. Please consider adding such analysis results. If you need more time to conduct this analysis, please let us know.

**Answer:** We thank for this comment. We analyzed the number of LRs in the pre-grown and newly grown root areas by fluridone and cotreatment of fluridone and retinal (Supplemental Fig. S14). In pre-grown root areas, fluridone increased LR number, and retinal cancelled this induction. In newly grown root areas, fluridone severely reduced LR number which has been described in Supplemental Fig. S3A. However, retinal partially rescued LR emergence under fluridone conditions. These results suggest that fluridone may regulate both PR growth and LR emergence via controlling retinal contents. On the other hand, we found that fluridone and NAA had less effect on LR growth compared to PR growth in both WT and *pds3-1* plants (Fig. 1C; Fig. 7B; Supplemental Fig. S14, C–E), suggesting the differential regulation of emerging-LR growth and PR growth. Supporting this hypothesis, the application of retinal did not alter the LR length treated by fluridone and NAA, or in *pds3-1* mutant (Supplemental Fig. S14, C–E).

**Comment- monitoring editor-1:**

Figure 8 nicely summarizes the proposed model differentiating short- and long-term regulation of PR growth mediated by carotenoids. I think you could indicate in this figure the contribution (novelty) of this study by highlighting the predictions that were derived from your experimental results (e.g. showing the corresponding dashed lines in red or in another color).

**Answer:** We thank for this comment. After revision, we highlight the change in the regulation pathway after fluridone treatment shown as Fig. 8. We use gray color and smaller font size to indicate inhibited contents and pathways, and larger font size to indicate increased contents and activated pathways. Red color indicates the key regulators in this process.

**Reviewer: 1**

**Comment-rev1-1:**

I agree with the authors response, noted here "For the partial bleaching (only newly emerged true leaves) phenotype, we consider the reason as short-term inhibition of carotenoid biosynthesis mainly targets newly synthesized carotenoids but has less effect on

the carotenoid pool." This hypothesis should be clearly explained in the manuscript itself, as it is very important to understanding the various phenotypes that the authors observe. I think this discussion should be introduced early in the paper (lines 134-135 perhaps) to make it clear that short-term fluridone treatment is expected to target newly synthesized carotenoids and apocarotenoids.

**Answer:** We thank for this comment greatly, and we understand it is quite important to describe and emphasize the phenotype of short-term fluridone treatment. So we added the introduction below "No such difference between cotyledons and true leaves was reported in norflurazon-grown or *psy* plants (Van Norman et al., 2014). This suggests that the short-term fluridone treatment may preferentially target developing organs." (line 134-137).

**Comment-rev1-2:**

Given the new data the authors present, they should test whether retinal can rescue the lateral roots in the newly grown tissue post-fluridone treatment. This experiment is important, because otherwise, it seems very strange that retinal affects root growth but not lateral root development, which are both regulated by auxin, especially given the results of previous work cited in the manuscript. It would also decrease the need to measure retinal levels, which the authors say is beyond the scope of this paper.

**Answer:** We thank for this comment. We analyzed the number of LRs in the pre-grown and newly grown root areas by fluridone and cotreatment of fluridone and retinal (Supplemental Fig. S14). In pre-grown root areas, fluridone increased LR number, and retinal cancelled this induction. In newly grown root areas, fluridone severely reduced LR number which has been described in Supplemental Fig. S3A. However, retinal partially rescued LR emergence under fluridone conditions. These results suggest that fluridone may regulate both PR growth and LR emergence via controlling retinal contents. On the other hand, we found that fluridone and NAA had less effect on LR growth compared to PR growth in both WT and *pds3-1* plants (Fig. 1C; Fig. 7B; Supplemental Fig. S14, C–E), suggesting the differential regulation of emerging-LR growth and PR growth. Supporting this hypothesis, the application of retinal did not alter the LR length treated by fluridone and NAA, or in *pds3-1* mutant (Supplemental Fig. S14, C–E).

**Reviewer: 2**

**Comment-rev2-1:**

A major concern raised by the two initial reviewers is the potential discrepancy between the role of retinal in LR responses observed in this study and the one reported in previously studies (Van Norman 2014 and Dickinson 2021). In the revision, the authors suggest that temporal differences between the studies-specifically, short-term inhibition versus permanent lack of carotenoids-may account for the discrepancies. They also observed that the fluoridone-induced enhancement of LR development is observed only in the preexisting root tissue, while newly form tissue exhibits opposite response. Although the authors provide new data to address the reviewers´ comments, this aspect still remains unclear in the manuscript. Previous findings indicate that retinal mediates lateral root initiation (Dickinson), but it is possible that retinal functions differentially in LR initiation versus emergence. This possibility could potentially explain some of the differences observed. However, the authors did not analyze whether the observed LR promotion results from elongation of the preexisting LR primordia or the development of new primordia. Additionally, while they report the complex response to fluridone treatment between the pre-existing and new grown root tissue, is not clear why LR roots are inhibited in the new tissues with respect to LR primordia initiation and emergence. A more detailed analysis, including in quantification of LR primordia during the treatments and the initiation events per cell number, would be highly informative and valuable in addressing and discussing the potential differences with the published literature.

**Answer:** We thank for this comment. We agree that it would be very valuable for strengthening the manuscript if we analyze LR primordia stage under fluridone and retinal treatment and it would be a good evidence in addressing the differences with Dickinson's paper. However, actually this paper is not a follow of Dickinson's paper and the aspects of discovery within these two papers are parallel (LR capacity vs. PR growth). It is unclear yet fluridone inhibits LR primordia formation or emergence in the newly grown root areas, but this effect of fluridone may be a reduction of root clock. It corresponds to the low LR formation in *pds3-1* and *psy* mutant, as well as the low LR capacity in seedlings grown on norflurazon-containing medium (Fig. 1G; Supplemental Fig. S1B) (Van Norman et al., 2014). We are sorry that the analysis of LR primordia may be warranted in the future.

**Van Norman JM, Zhang J, Cazzonelli CI, Pogson BJ, Harrison PJ, Bugg TD, Chan KX, Thompson AJ, Benfey PN** (2014) Periodic root branching in *Arabidopsis* requires synthesis of an uncharacterized carotenoid derivative. *Proceedings of the National Academy of Sciences, USA* **111:** E1300-E1309

**Comment-rev2-2:**

Another aspect requested by the reviewers was evidence for the decrease of retinal levels under the analyzed conditions. The authors argue that quantifying retinal levels is inherently difficult, which is understandable. However, this remains a critical point to conclusively demonstrate the participation of this apocarotenoid in the observed response. The authors note a substantial reduction in various carotenoids 3 days after fluoridone treatment (Figure S4), which could influence the levels of diverse apocarotenoid molecules, not only retinal. While the authors provide evidence that neither ABA, strigolactones, nor β-cyclocitral alone seem to contribute to the observed responses (as neither of them complement the observed phenotypes), in contrast to retinal, supporting the latter as a potential effector of these responses. Nevertheless, given the diversity of apocarotenoids produced (many of unknown structures and functions), it is important for the authors to consider these findings when drawing conclusions and for a more comprehensive discussion.

**Answer:** We thank for this comment. It is well known for the variety of apocarotenoids. Recent years many bioactive apocarotenoids as root regulators are identified (e.g., β-cyclocitral, anchorene and iso-anchorene) (Dickinson et al., 2019; Jia et al., 2019; Jia et al., 2021). This is the reason why we employed several apocarotenoids (i.e., ABA, strigolactones, β-cyclocitral and retinal) to examine their roles in root growth regulation under fluridone conditions. Although maybe there are other apocarotenoids responsible for fluridone-mediated PR growth suppression, in this study we demonstrated that at least retinal is the apocarotenoid that rescues the root growth suppression caused by fluridone. For other apocarotenoids, we excluded their participation in fluridone-mediated root growth regulation described in Results (line 170-171, 278-279).

**Dickinson AJ, Lehner K, Mi J, Jia K, Mijar M, Dinneny JR, Al-Babili S, Benfey P** (2019) β-Cyclocitral is a conserved root growth regulator. *Proceedings of the National Academy of Sciences, USA* **116:** 10563-10567

**Jia K, Dickinson A, Mi J, Cui G, Xiao T, Kharbatia N, Guo X, Sugiono E, Aranda M, Blilou I, Rueping M, Benfey P, Al-Babili S** (2019) Anchorene is a carotenoid-derived regulatory metabolite required for anchor root formation in *Arabidopsis*. *Science Advances* **5:** eaaw6787

**Jia KP, Mi J, Ablazov A, Ali S, Yang Y, Balakrishna A, Berqdar L, Feng Q, Blilou I, Al-Babili S** (2021) Iso-anchorene is an endogenous metabolite that inhibits primary root growth in Arabidopsis. *The Plant Journal* **107:** 54-66

**Comment-rev2-3:**

Although is not fully clear whether the quantification of the different carotenoids was performed with the whole seedlings or only root tissue, these data indicate that the fluoridone treatment results in significant changes in the overall carotenoid pool in the plant. It is unclear why the authors conclude fluridone mainly targets newly synthesized carotenoids, as stated in the reviewer´s response letter.

**Answer:** We thank for this comment. The quantification of different carotenoids was conducted on the whole seedlings (Supplemental Fig. S4, B–G). We raise this hypothesis based on the phenotype that newly emerged true leaves were firstly bleached by fluridone (Supplemental Fig. S4A), at the same time, cotyledons showed chlorosis and then generally turned white (data not shown). It is very hard to consider that the reduction in newly synthesized carotenoids and the carotenoid pool is of an equivalent extent following fluridone treatment. If true, it is hard to explain the difference between the complete bleaching of newly emerged true leaves and the chlorosis of cotyledons. Thus we consider that short-term fluridone treatment may mainly target newly synthesized carotenoids.

**Comment-rev2-4:**

As suggested by the reviewers, the authors analyzed the LR response mediated by fluridone in psy and pds3 mutants, strengthening this study. They observed that both psy and pds3 mutants exhibit PR length and fewer LR number compared to WT, consistent with previous reports. Interestingly, the psy mutant responds to the short-term NF treatment by displaying shorter PR and more LRs, but this response is not observed in the pds3 mutant. Moreover, they also found that the addition of retinal promotes PR growth in pds3. From these results, the authors concluded that PDS activity is required for root responses. However, it remains unclear how these results led the authors to conclude that a transient block of retinal is responsible for PDS-mediated root regulation. Specifically, how do the authors explain that a carotenoid/retinal transient block mediated by PDS occurs in the psy1 mutant that lacks carotenoids due to the PSY mutation? This aspect requires further clarification. Additionally, the analyses of the DR5 content after fluoridone treatment in the psy mutant, could be very informative data.

**Answer:** We thank for this comment. One explanation for *psy* mutant response to fluridone is that *psy* mutant used in this study may not be a null mutant. The T-DNA insertion site of the

*psy* mutant (SALK_054288) retained three of the eighteen amino acids constituting the substrate binding pocket, two of the fourteen amino acids putatively constituting the catalytic moiety, and one of the two active site lids (Zhai et al., 2016) (Supplemental Fig. S15). To prove this hypothesis, the quantification of phytoene contents in *psy* mutant is necessary in the future. We added related discussion in the manuscript (line 389-398).

**Zhai S, Li G, Sun Y, Song J, Li J, Song G, Li Y, Ling H, He Z, Xia X** (2016) Genetic analysis of phytoene synthase 1 (*Psy1*) gene function and regulation in common wheat. *BMC Plant Biology* **16:** 228

**Comment-rev2-5:**

Although this point was not raised by the reviewers, it is important to consider the diverse reports demonstrating a significant role of PDS3 in maintaining the plastoquinone pool and generating GUN-mediated retrograde plastid signals (Pfannschmidt et al., 2009, Aluru et al., 2009, Rosso et al., 2009). In fact, NF has been shown to alter the redox balance of the PQ pool (Brausemann et al., 2017). How do these PDS roles fit with the findings of this study? This is a relevant aspect that merits consideration throughout the manuscript, as the data in this study strongly highlight the importance of the PDS activity. Additionally, it has been shown that the genes affected in the pds3 and NF-treated plants are not equivalent (Fourdree 2010). For example, many genes related to gibberellins are exclusively affected in pds3, explaining the dwarf phenotype observed in this mutant. Moreover notable similarities in the expression were observed between NF-treated plants and the immutans mutant that encodes an alternative oxidase (Aluru 2009).

**Answer:** We thank for this comment. Indeed, we demonstrate that fluridone-mediated root growth regulation is dependent on the PDS activity (i.e., line 158-159). We do not deny the potential roles of *PDS3* in retrograde signaling for regulating root growth. Retrograde signaling is highly complex which affects a large set of gene expression as shown in previous research (Aluru et al., 2009). Considering the length and focus of manuscript, it is hard to clarify the participation of retrograde signaling in fluridone-mediated root growth regulation in this study. We also agree that the genes affected in *pds3* mutant and fluridone-treated plants are not equivalent, since they have different response to retinal (Supplemental Fig. S16). We also do not deny the possibility of gibberellin acid for regulating plant development (including root development), since exogenous application of gibberellin acid could rescue the dwarf phenotype of *pds3* mutant (Qin et al., 2007). However, in this study we investigate new findings that the apocarotenoid retinal, as a

downstream substrate of carotenoids, regulates PR growth via the auxin pathway. We focus on this issue and perform a series of experiments. Although it is clearly stated about the similarities between NF-treated plants and the immutans mutant, it is also stated that their molecular phenotypes are not identical, which may be due to differences in the mechanism of inhibition of PDS in the two tissue types (Aluru et al., 2009). Thus we consider it is a little bit difficult to link fluridone-treated plants and immutans mutant within the frame of this study.

**Qin G, Gu H, Ma L, Peng Y, Deng XW, Chen Z, Qu LJ** (2007) Disruption of phytoene desaturase gene results in albino and dwarf phenotypes in *Arabidopsis* by impairing chlorophyll, carotenoid, and gibberellin biosynthesis. *Cell Research* **17:** 471-482

**Aluru MR, Zola J, Foudree A, Rodermel SR** (2009) Chloroplast photooxidation-induced transcriptome reprogramming in Arabidopsis *immutans* white leaf sectors. *Plant Physiology* **150:** 904-923

**Comment-rev2-6:**

Line 258. As previously noted by a reviewer, is intriguing that fluridone treatment, there is a significantly induces the expression of the CCD7 and CCD8 genes (Fig 6C and D). The authors suggest that this might be a feedback response. However, these genes encoded proteins involved in carotenoid catabolism and in the synthesis strigolactones and other apocarotenoids, possibly including retinal. A comment in this would be important. It might also be interesting to analyze whether the transient response to fluridone observed here is altered in ccd8 or ccd7 single or double mutants.

**Answer:** We thank for this comment. The induction of expression in *CCD7* and *CCD8* by fluridone here was as the evidence that fluridone-induced auxin contents may feedback to accelerate carotenoid catabolism. We cannot deny that it will be interesting to see the response of *ccd7* and *ccd8* single and double mutant to fluridone. However, *max3/ccd7* used in this study showed WT-like resistance to fluridone (Supplemental Fig. S5C). In fact, the retinal biosynthesis pathway in plants is still unknown, and this study does not focus on investigating the enzymes and genes involved in retinal metabolism but discovering the new aspect of retinal in regulating root development.

**Comment-rev2-7:**

A major piece of evidence supporting the role of retinal in fluoridone-induced root architectural changes is the observed partial reversion of the fluoridone-mediated PR

growth inhibition and LR formation induction. However, it remains unclear whether the inhibition of LR in the new formed tissue is also reverted? Additionally, what is the fluridone-mediated phenotype in the psy mutant with the addition of retinal?

**Answer:** We thank for this comment. We analyzed the number of LRs in the pre-grown and newly grown root areas by fluridone and cotreatment of fluridone and retinal (Supplemental Fig. S14). In pre-grown root areas, fluridone increased LR number, and retinal cancelled this induction. In newly grown root areas, fluridone severely reduced LR number which has been described in Supplemental Fig. S3A. However, retinal partially rescued LR emergence under fluridone conditions. These results suggest that fluridone may regulate both PR growth and LR emergence via controlling retinal contents. On the other hand, we found that fluridone and NAA had less effect on LR growth compared to PR growth in both WT and *pds3-1* plants (Fig. 1C; Fig. 7B; Supplemental Fig. S14, C–E), suggesting the differential regulation of emerging-LR growth and PR growth. Supporting this hypothesis, the application of retinal did not alter the LR length treated by fluridone and NAA, or in *pds3-1* mutant (Supplemental Fig. S14, C–E). In this study we did not check the phenotypes of *psy* mutant treated by retinal, since it has shown the different response to fluridone within *pds3* and *psy* mutant (Fig. 1, F and G; Supplemental Fig. S1, A and B). The reason why *psy* mutant still has sensitivity to fluridone is explained in "Comment-rev2-4".

**Comment-rev2-8:**

As mentioned by the reviewer, the level of retinal used in this study is 20 times higher than in previous reports. The authors demonstrate that this high retinal level did not affect root growth, but the conditions used for the retinal treatment analysis need to be clearly described.

**Answer:** We thank for this comment. The conditions used for retinal treatment are under continuous light (80–110 µmol $m^{-2}$ $s^{-1}$) (Materials and Methods line 470-471). Besides, in bioluminescence imaging analysis the light condition is red and blue light (100 µmol $m^{-2}$ $s^{-1}$) (Materials and Methods line 520)

**Comment-rev2-9:**

The authors propose that the difference in root growth between fluridone-treated and pds3-1 mutant may reflect process of plastid degradation versus undifferentiated plastids (lines 396-399). Are the authors suggesting that fluridone promotes degradation of the

root plastids? Do the authors have any evidence in this regard or published data that supports this statement?

**Answer:** We thank for this comment. Previous research shows when fluridone solution is applied to the 40% growth of the 3rd leaf of maize, the leaf grows into a white/green mixed leaf: complete white in basal part (W), pale green in the middle (PG) and green in the apical (G) (Kim et al., 2004). This paper demonstrates that in the developed leaves, the cell damage caused by fluridone results from the cell destruction by excessive light-induced photo-oxidation; however, in the developing and undifferentiated tissues, the cell damage may be more dependent on a loss of photosystem followed by carbohydrate deficiency (Kim et al., 2004). Another reference also shows that applying NF solution to the leaves of 5-day-old Arabidopsis seedlings causes leaf chlorosis (Kim and Apel, 2013). They also show a loss of chloroplast integrity by NF solution treatment. On the other hand, the chloroplasts of seedlings grown on NF-containing medium do not lose their protein import capacity and remain intact. Overall, these research provide evidence that short-term fluridone treatment in this study may lead to plastid damage or degradation.

**Kim JS, Yun BW, Choi JS, Kim TJ, Kwak SS, Cho KY** (2004) Death mechanisms caused by carotenoid biosynthesis inhibitors in green and in undeveloped plant tissues. *Pesticide Biochemistry and Physiology* **78:** 127-139

**Kim C, Apel K** (2013) $^1$O2-mediated and EXECUTER-dependent retrograde plastid-to-nucleus signaling in norflurazon-treated seedlings of *Arabidopsis thaliana*. *Molecular Plant* **6:** 1580-1591

**Comment-rev2-10:**

The role of PDS as a carotenoid transporter is still under debate.

**Answer:** We thank for this comment. PDS proteins may be involved in the trafficking route of carotenoid transport from the synthesis sides (envelope membranes) to the functional sides (thylakoid membranes) (Lopez et al., 2008). We raise this hypothesis since we consider that the carotenoids in biosynthesis sides and transport routes but not in functional sides are preferentially inhibited by fluridone. However, we did not discuss this issue in the manuscript since we consider it is imagination and needs more evidence to support.

**Lopez AB, Yang Y, Thannhauser TW, Li L** (2008) Phytoene desaturase is present in a large protein

complex in the plastid membrane. *Physiologia Plantarum* **133:** 190-198

03-Jul-2024
Prof. Dr. Masaaki K. Watahiki
Hokkaido University
Hokkaido University
Sapporo
Japan

MSID: PP2024-RA-00788DR1
MS TITLE: The exogenous application of the apocarotenoid retinaldehyde negatively regulates auxin-mediated root growth

Dear Dr. Masaaki Watahiki:

I am pleased to inform you that your revised manuscript "The exogenous application of the apocarotenoid retinaldehyde negatively regulates auxin-mediated root growth" has been accepted for publication in Plant Physiology. Your manuscript will be checked for consistency with journal formatting and image standards by one of our Science Editors, who will contact you shortly. They may have a number of queries and suggestions for you. Please keep in mind that they work to increase the online visibility of your article and its attraction for readers from the widest of backgrounds. You will have the opportunity to review any revisions, and I hope you will work constructively with them to get the most out of your research. You will also have the opportunity to review all changes when you receive the author proofs for your article.

Once validated for publication, your article will be sent to a Science Editor for a final review of your manuscript and source files. After your final files are uploaded, your article will appear in the next available issue and the online Preview version of your article will be posted after your signed Copyright and Checklist forms and final source files are received.

Please be sure to include the following in your manuscript file:
Harvard style citations
List of all author contributions and funding information at the beginning of manuscript after the titles and author list.
List all supplemental materials associated with the article, with titles, at the end of the ms, before the references and acknowledgments section.
Complete Figure Legends for all figures appearing in the article listed before the Literature Cited.

Thank you and congratulations on your Plant Physiology paper!

Sincerely,
Editor, Plant Physiology

=====
FIRST AUTHOR PROFILES
=====

We recognize the hard work that goes into being First Author of a Plant Physiology paper by publishing a "First Author Feature" for each First Author alongside your paper.

If the first author(s) would like to submit information for a feature on Facebook and Twitter, fill out the form by clicking on the link below:

Plant Physiology First Author Feature Form: https://forms.office.com/r/ZBCGZfhm8F
If you cannot access the form through the link above, please contact Rachel at rbelsky@aspb.org.

To upload an image of yourself to accompany your profile, please put your photo in the correct journal folder at the link below. You must label your image: Author First Name Last Name Article Title.

First Author Profile Image Dropbox: https://www.dropbox.com/scl/fo/xz029p7qjr2jml2rp2kmq/h?dl=0&rlkey=6fkxcaqmxx35ap152b2osy1fz

=====

IMPORTANT REMINDER: PEER REVIEW REPORTS
=====

If you opted to publish a peer review report along with your article during the original submission process, it will be prepared by the editorial staff and publicly posted with your manuscript, inside the zip file that contains any other supplemental material. As a reminder, the peer review report is a public record of all comments from editors and reviewers, as well as your prior responses, as you received them in the decision letters for each draft of your manuscript. If you agreed to publish this report and have changed your mind, or are not sure if you selected this option, please contact the editorial office as soon as possible before signing the license agreement from our publisher.

------------------------------------------------------------------------------ Reviewer comments:
